# What cervical screening is appropriate for women who have been vaccinated against high risk HPV? A simulation study

**DOI:** 10.1002/ijc.31094

**Published:** 2017-11-10

**Authors:** Rebecca Landy, Peter Windridge, Matthew S. Gillman, Peter D. Sasieni

**Affiliations:** ^1^ Centre for Cancer Prevention, Wolfson Institute of Preventive Medicine, Barts and The London School of Medicine Queen Mary University of London, Charterhouse Square London EC1M 6BQ UK

**Keywords:** simulation, HPV, cervical cancer, vaccination, screening, policy

## Abstract

Women vaccinated against HPV16/18 are approaching the age for cervical screening; however, an updated screening algorithm has not been agreed. We use a microsimulation model calibrated to real published data to determine the appropriate screening intensity for vaccinated women. Natural histories in the absence of vaccination were simulated for 300,000 women using 10,000 sets of transition probabilities. Vaccination with (*i*) 100% efficacy against HPV16/18, (*ii*) 15% cross‐protection, (*iii*) 22% cross‐protection, (*iv*) waning vaccine efficacy and (*v*) 100% efficacy against HPV16/18/31/33/45/52/58 was added, as were a range of screening scenarios appropriate to the UK. To benchmark cost‐benefits of screening for vaccinated women, we evaluated the proportion of cancers prevented per additional screen (incremental benefit) of current cytology and likely HPV screening scenarios in unvaccinated women. Slightly more cancers are prevented through vaccination with no screening (70.3%, 95% CR: 65.1–75.5) than realistic compliance to the current UK screening programme in the absence of vaccination (64.3%, 95% CR: 61.3–66.8). In unvaccinated women, when switching to HPV primary testing, there is no loss in effectiveness when doubling the screening interval. Benchmarking supports screening scenarios with incremental benefits of ≥2.0%, and rejects scenarios with incremental benefits ≤0.9%. In HPV16/18‐vaccinated women, the incremental benefit of offering a third lifetime screen was at most 3.3% (95% CR: 2.2–4.5), with an incremental benefit of 1.3% (−0.3–2.8) for a fourth screen. For HPV16/18/31/33/45/52/58‐vaccinated women, two lifetime screens are supported. It is important to know women's vaccination status; in these simulations, HPV16/18‐vaccinated women require three lifetime screens, HPV16/18/31/33/45/52/58‐vaccinated women require two lifetime screens, yet unvaccinated women require seven lifetime screens.

## Abstract 2

**What's new?**

In England, the first cohorts of teenage girls to be vaccinated against human papillomavirus (HPV) will soon be entering the country's cervical cancer screening program. However, appropriate screening intervals for vaccinated women are yet to be established. Here, the necessary number of lifetime screens for HPV16/18‐vaccinated women and HPV16/18/31/33/45/52/58‐vaccinated women was determined using the proportion of cancers prevented per screen benchmarked against current screening practice. The findings suggest that three lifetime screens would be sufficient for cervical cancer prevention in HPV16/18‐vaccinated women, while just two lifetime screens would be effective for HPV16/18/31/33/45/52/58‐vaccinated women.

Human papillomavirus (HPV) is necessary for the development of cervical cancer, with HPV types 16 and 18 known to be particularly high risk.^1^ There are two public health interventions to prevent cervical cancer: cervical screening and HPV vaccination. Cervical screening aims to identify and treat precancerous changes to the cervix, preventing cervical cancer developing. Current screening guidelines in England are for 3‐yearly cytology screening with HPV triage for women aged 25–49 and 5‐yearly for ages 50–64. HPV triage is carried out on cytology results which are borderline or mild (using the British Society for Colposcopy and Cervical Pathology terminology). In January 2016 the UK National Screening Committee announced their intention to introduce primary HPV testing. Randomised controlled trials show this is more effective in preventing cervical cancer.^2^ It is more sensitive than cytology for detecting high‐grade CIN, though less specific,^3^ and several countries are in the process of switching to primary HPV testing, with primary HPV testing introduced in the Netherlands in January 2017.

HPV vaccination of teenage girls became widespread in 2007–2009. In England, the HPV vaccine was introduced in 2008 for girls aged 12–13. In 2015, a nonavalent vaccine was approved for use in Europe, additionally protecting against HPV types 31/33/45/52/58; together with HPV types 16/18, they are responsible for approximately 90% of cervical cancers. The difference in screening offered may differ following vaccination with the bivalent (HPV types 16/18, responsible for approximately 70% of cervical cancers) or quadrivalent (HPV types 6/11/16/18, responsible for approximately 70% of cervical cancers and 90% of genital warts) and the nonavalent vaccines; this difference should be taken into account when estimating cost‐effectiveness.

Women vaccinated through the catch‐up programme (vaccinated aged 13–18 in 2009) first entered the screening programme in England in September 2016. Following the same screening intervals as unvaccinated women, as currently recommend in the U.S.,^4^ is unlikely to be a good use of resources. However, an updated algorithm has not yet been settled on. We do not directly address screening intensity in women vaccinated as part of the catch‐up cohort, as we consider women vaccinated at age 12, with the assumption that this is prior to HPV exposure; however the first full cohorts vaccinated against HPV16/18 approaching the recommended age for screening to begin, updated screening interval recommendations are required soon. If the screening programme continues to start at age 25, women vaccinated at age 12 will enter the screening programme from 2021.

Simulation models are increasingly used to inform health policy decisions, where information about long‐term outcomes will not be available for many years.^5^ Typically, models are calibrated to a particular population and thus provide simulation results specific to that population. Here, we instead investigate outcomes shown to be robust under diverse model calibrations and study their values and variation when model parameters are picked randomly from a realistic distribution. Thus our results should be applicable to a wide variety of populations.

## Materials and Methods

### Natural history model

**Figure 1:**
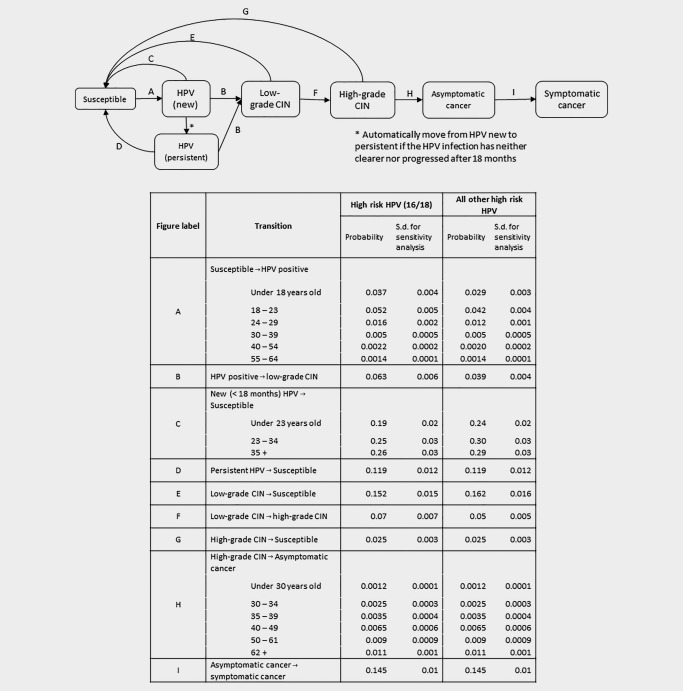
Possible transitions in the model, with six‐monthly transition probabilities for the natural history model. HPV‐16/18 and other (non‐16/18) high‐risk HPV processes are run separately.

We developed a simple model of cervical cancer natural history where transitions between disease states are made at half‐yearly intervals, from ages 12 to 80. Initially everyone is assumed to be HPV‐negative, and we do not allow for hysterectomies or deaths prior to age 80. The states and possible transitions are shown in Figure 1; “asymptomatic” refers to cancers only diagnosed as a result of screening, and “symptomatic” cancers are diagnosed without (or despite) screening. If an asymptomatic cancer is not detected through screening, it may progress to become symptomatic, according to the transition probabilities, or remain asymptomatic until age 80. In line with scientific evidence,^6^, ^7^ we assume cervical cancer cannot occur without HPV infection.

The transition probabilities are HPV‐type and age dependent. For simplicity, we consider two groups of HPV types: 16/18 and all other (non‐16/18) high‐risk types. As the transition probabilities are subject to uncertainty, 10,000 sets of normally distributed parameters were sampled independently, with means and standard deviations chosen to reflect the uncertainty reported in the literature (Fig. 1). We rejected parameter sets for which the simulation results were inconsistent with the literature (Table 1). This rejection may be considered to be a minimal version of calibration. Rather than calibrating to a particular population, we calibrate so the model is similar to a real population. To demonstrate the variability due to the simulations rather than the choice of parameters, we also simulate 1,000 cohorts using fixed parameters (i.e., setting the standard deviation to 0). Each cohort consists of 300,000 simulated natural histories, the approximate number of girls aged 12 in England.^8^ Confidence intervals were based on empirical limits from the 10,000 cohorts with randomly selected parameters.

**Table 1:** Rejection criteria for sampled model parameters, applied to 300,000 simulated natural histories, in the absence of vaccination or screening. Parameter sets were rejected if any value was below the lower limit or above the upper limit

Criteria	Lower limit	Upper limit
Proportion abnormal (i.e., HPV positive, CIN or cancer)		
at age 22	0.200	0.466
at age 32	0.108	0.176
at age 42	0.063	0.103
at age 52	0.043	0.078
at age 62	0.037	0.069
Proportion of the population who develop cancer		
by age 30	0.000135	0.00344
during ages 30–50	0.002185	0.01138
by age 80	0.01093	0.030985
Overall proportion of cancers that are HPV16/18	0.65	0.76

**Figure 2:**
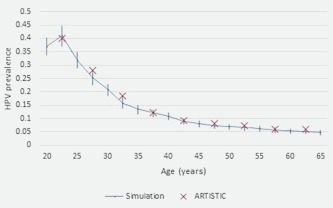
Mean simulated HPV prevalence by age, and observed HPV prevalence by age in the ARTISTIC trial.^9^ [Color figure can be viewed at wileyonlinelibrary.com]

Proportion abnormal: lower limits from Kaiser Permanente Northern California for ages 32–62, no data were available for age 22.^29^ Upper limit from the HPV pilot study in England, using data from Sheffield, which was the site with the highest HPV positivity (Fig. 2).^30^

Proportion of the population who develop cancer: lower limit: Finland in 1972–1976. Upper limit: Brazil in 1973.^12^

Overall proportion of cancers that are HPV16/18: meta‐analysis.^11^

We did not consider new HPV infections beyond age 65; they are assumed to be rare, and, due to the slow development of cervical cancer, are unlikely to result in cancer. We calibrated age‐specific HPV prevalence to the published age‐specific HPV prevalence data from the ARTISTIC trial in England (Fig. 2) using an iterative process to estimate the transition probabilities,^9^ with estimates in the literature as a starting point.^10^ We ensured an appropriate proportion of cancers were caused by HPV16/18,^11^ and the range of lifetime risks of cervical cancer in the absence of screening was realistic.^12^

The model was run independently for HPV16/18 and non‐16/18 high‐risk HPV types. The results were combined by taking the more advanced state at each time point (e.g., low‐grade CIN is more advanced than HPV infection). We assumed that 10% of the population were high risk – i.e., they had a higher risk of becoming infected with both HPV‐16/18 and non‐16/18 high‐risk HPV types; this was modeled by increasing the ‘susceptible’ to ‘HPV infection’ transition probability reported in Figure 1 by 20% for these individuals.

### Vaccination

In addition to the natural history in the absence of HPV vaccination, five scenarios exploring vaccine efficacy were considered. In all scenarios, 100% vaccination coverage was assumed to occur at age 12, as we are interested in screening algorithms for women known to have been vaccinated.

1. The vaccine prevents 100% of HPV16/18 infections, responsible for around 70% of cervical cancers^1^, ^13^; the protection does not wane with time, but no cross‐protection against other (non‐16/18) high‐risk HPV infections associated with cervical cancer is achieved.
2. The quadrivalent vaccine provides some cross‐protection, preventing 14.7% of non‐16/18 high‐risk HPV infections associated with cervical cancer, in addition to 100% of HPV16/18 infections. The protection does not wane with time.
3. The bivalent vaccine provides some cross‐protection, preventing 22.1% of non‐16/18 high‐risk HPV infections associated with cervical cancer, in addition to 100% of HPV16/18 infections. The protection does not wane with time.
4. The vaccine prevents 100% of HPV16/18 infections at vaccination (age 12), but the effectiveness drops by 0.25% (absolute) per six months since vaccination, making the vaccine less effective, so by age 27, the protection is 92.5% for HPV16/18. There is no cross‐protection.
5. The nonavalent vaccine: 63.6% of non‐16/18 high‐risk HPV infections associated with cervical cancer are prevented,^14^ in addition to 100% of HPV16/18 infections. The protection does not wane with time.

The level of cross‐protection for the quadrivalent and bivalent vaccines (II and III above) were estimated from a weighted average of the level of protection against 6‐month type‐specific HPV persistence in trials of the quadrivalent^15^ and bivalent^16^ vaccine, weighted by HPV type prevalence in cancers not caused by HPV16/18^1^; see Supporting Information. Waning vaccine effectiveness was based on 95.6% effectiveness after 9.4 years against incident infection.^17^ The vaccines only affect the transition between susceptible and HPV positive.

### Screening and treatment

Various screening programmes were applied to the simulated natural history datasets. We considered both 100% compliance with screening, to generate the optimal recommendations for women who adhere to screening recommendations, and a more realistic scenario, based on cervical screening attendance in England (Supporting Information), to provide population‐level recommendations. The screening scenarios considered are given in Table 2.

**Table 2:** Screening scenarios considered in the simulation study

Scenario	Number of lifetime screens	Screening ages
A	12	25, 28, 31, 34, 37, 40, 43, 46, 49, 52, 57, 62
B	7	25, 31, 37, 43, 49, 55, 65
C	4	30, 40, 50, 65
D	4	25, 35, 50, 65
E	3	30, 40, 55
F	3	30, 45, 60
G	3	25, 45, 65
H	2	30, 45
I	2	30, 55
J	1	30
K	1	35
L	1	40
M	1	45

A′ and B′ as A and B, but with cytology as the primary screening test. Scenarios A–D were considered in unvaccinated and vaccinated cohorts. Scenarios E–M were only considered in vaccinated cohorts.

All screening scenarios assume HPV primary testing with partial genotyping for HPV16/18 (to allow differential follow‐up) with cytology triage. For details of the assumptions regarding test sensitivity, see Supporting Information. We additionally consider two screening scenarios (A′, B′) under the current screening programme, using cytology with HPV triage. These scenarios mirror screening scenarios A and B in Table 2, but use cytology as the primary test, with HPV triage of low‐grade cytological abnormalities. Cancer is diagnosed when transition occurs from asymptomatic to symptomatic cancer (i.e., assuming no delay in symptom recognition), or through screening. Therefore, in the absence of screening only symptomatic cancers are diagnosed.

### Statistical methods

We report the mean number of screens, including recall tests following a positive HPV test and negative cytology test, but excluding tests taken during surveillance following treatment. We compare the number of cancers that would be diagnosed under the vaccination/screening scenarios to the number of symptomatic cancers in the absence of screening or vaccination (asymptomatic cancers would not be diagnosed in the absence of screening), and from these numbers calculate the proportion of cancers that would be prevented by that combination of vaccination and screening. Rather than using traditional measures of cost‐effectiveness, we calculate the incremental benefit of each additional screen as the difference in the proportion of cancers prevented with a given number of screens to the proportion of cancers prevented using one fewer screen (when multiple scenarios with the same number of lifetime screens are considered, we use the scenario which prevents the highest proportion of cancers), and dividing this by the difference in number of lifetime screens between the two scenarios (which will be close to but not exactly 1, due to differences in the number of follow‐up tests between scenarios). For example, if scenario A prevents 85% of cervical cancers using 3.15 lifetime screens, and scenario B prevents 89% using 4.20 lifetime screens, the incremental benefit per additional screen is (89 − 85)/(4.20 − 3.15), 3.8%. The incremental benefit of the current screening practice (12 lifetime screens) is taken to be the benefit of each additional screen compared to 6‐ and 10‐yearly screening (7 lifetime screens). We use 2.5th and 97.5th percentiles to provide empirical 95% central ranges (95% CR) for the percentage of cancers prevented and incremental benefit.

We calculated a benchmark for what level of incremental benefit would be required for a more intensive screening programme to be accepted by calculating the incremental benefit of 3(5)‐yearly HPV testing compared to 6(10)‐yearly HPV testing, and 3(5)‐yearly cytology testing compared to 6(10)‐yearly cytology testing, each with realistic screening coverage. Incremental benefits greater than that achieved by 3(5)‐yearly cytology are assumed to be worthwhile. Incremental benefits less than that achieved by 3(5)‐yearly HPV testing are assumed not to be acceptable. We do not attempt to identify an exact threshold.

The simulation model was coded in C++, and the data analysis was carried out in Stata v13.1.

## Results

The main results use 10,000 parameter sets; full results for all vaccination/screening scenarios considered are presented in Supporting Information, Tables S1–S4. In the absence of screening, with no vaccination, the cumulative risk of cervical cancer to age 80 was 2.19% (95% CR: 1.67–2.69). The vaccine which protected against 100% of HPV16/18 with no waning prevented 70.3% of cervical cancers (95% CR: 65.1–75.5), the quadrivalent vaccine with cross protection prevented 74.0% (95% CR: 69.5–78.6), the bivalent vaccine with cross protection prevented 76.4% (95% CR: 72.2–80.6), the vaccine with waning efficacy prevented 65.8% of cancers (95% CR: 60.8–70.8) and the nonavalent vaccine prevented 88.4% (95% CR: 86.1–90.5).

In the absence of vaccination, the incremental benefit (per screen) of 3(5)‐yearly screening with cytology compared with 6(10)‐yearly screening was 2.0% of cervical cancers that would occur in the absence of any vaccination or screening (95% CR: 1.2–2.8), assuming realistic screening coverage. The corresponding incremental benefit for 3(5)‐yearly HPV testing in the absence of vaccination compared to 6(10)‐yearly screening with realistic screening coverage was 0.9% (95% CR: 0.3–1.7). We use these as a range of benchmarks for screening in vaccinated women, rejecting incremental benefits ≤0.9% and accepting incremental benefits ≥2.0% of cancers prevented per additional screen.

With realistic HPV primary screening coverage, in the absence of vaccination, the incremental benefit per screen of having 6‐ and 10‐yearly screening compared with 4 lifetime screens would be a 3.0% (95% CR: 2.2–3.8) reduction in cervical cancers per additional screen (Table 3). With full screening coverage, the benefits per additional screen are very similar.

**Table 3:** Mean number of screens, reduction in cancer incidence and incremental benefit (the reduction in cancer incidence per additional screen) for 100% and realistic screening coverage, from 10,000 simulated datasets of 300,000 women with natural history parameters drawn from the distributions given in Figure 1

		100% screening coverage			Realistic screening coverage		
Vaccine	Screening scenario	Mean number of screens	% reduction in cancers compared to no vaccination and no screening (95% CR)	% incremental benefit per screen (95% CR)	Mean number of screens	% reduction in cancers compared to no vaccination and no screening (95% CR)	% incremental benefit per screen (95% CR)
None	None	Lifetime risk: 2.2% (95% CR: 1.7%–2.7%)			Lifetime risk: 2.2% (95% CR: 1.7%–2.7%)		
	*Cytology primary testing* a						
	6/10 yearly^b^	7.0	78.7 (75.2–81.6)	11.2 (10.7–11.7)^e^	4.6	60.3 (57.0–63.1)	13.1 (12.4–13.7)^e^
	3/5 yearly^c^	12.0	86.5 (83.8–88.7)	1.6 (1.3–1.8)	6.6	64.3 (61.3–66.8)	2.0 (1.2–2.8)
	*HPV primary testing* d						
	30, 40, 55	3.4	75.5 (71.5–78.5)	22.2 (21.0–23.3)^e^	2.7	62.1 (58.6–64.9)	23.1 (21.8–24.3)^e^
	30, 40, 50, 65	4.4	78.4 (74.8–81.1)	3.0 (2.0–4.1)	3.2	63.8 (60.4–66.4)	3.1 (0.5–5.8)
	6/10 yearly^b^	7.5	86.7 (84.0–88.8)	2.6 (2.2–3.1)	5.1	69.3 (66.5–71.5)	3.0 (2.2–3.8)
	3/5 yearly^c^	12.7	90.9 (88.9‐ 92.5)	0.8 (0.6–1.0)	7.2	71.2 (68.7–73.2)	0.9 (0.3–1.7)
HPV16/18	None		70.3 (65.1–75.5)			70.3 (65.1–75.5)	
	30, 45	2.2	89.6 (87.3–91.7)	3.9 (2.9–5.0)	1.7	86.0 (83.2–88.6)	3.9 (2.6–5.2)
	30, 40, 55	3.2	92.4 (90.5–94.0)	2.7 (1.9–3.5)	2.5	88.3 (85.9–90.6)	2.8 (1.7–3.9)
	30, 40, 50, 65	4.2	93.4 (91.6–94.8)	1.0 (0.4–1.6)	3.1	88.9 (86.6–91.1)	1.0 (−0.3–2.5)
Nonavalent	None		88.4 (86.1–90.5)			88.4 (86.1–90.5)	
	35	1.0	94.3 (93.0–95.5)	5.8 (4.6–7.1)	0.9	93.4 (92.0–94.7)	5.7 (4.5–7.0)
	30, 45	2.1	95.9 (94.8–96.9)	1.6 (1.0–2.1)	1.6	94.5 (93.3–95.7)	1.5 (0.8–2.3)
	30, 40, 55	3.1	97.0 (96.1–97.7)	1.0 (0.6–1.5)	2.5	95.4 (94.3–96.4)	1.1 (0.5–1.7)

a Primary cytology testing with HPV triage.

b Six‐yearly screening from ages 25–49 years, 10‐yearly screening from ages 50–64 years.

c Three‐yearly screening from ages 25–49 years, 5‐yearly screening from ages 50–64 years.

d Primary HPV testing with cytology triage.

e Compared to no screening.

The proportion of cancers prevented is slightly higher for women vaccinated against HPV16/18 (scenario I) but not screened (70.3% (95% CR: 65.1–75.5)) and 3(5)‐yearly cytology screened unvaccinated women with realistic screening coverage (64.3% (95% CR: 61.3–66.8)).

We next consider the vaccine with 100% protection against HPV16/18 with neither waning nor cross‐protection, with realistic HPV primary screening compliance at different screening intervals (Table 4). In the scenario of two lifetime screens, the incremental benefit per screen in vaccinated women was the prevention of an additional 3.9% (95% CR: 2.6–5.2) of cancers compared to the scenario with one lifetime screen (Table 4). With three lifetime screens, depending on the ages at which they occurred, the incremental benefit of the third screen was around 2% (Table 4). There was no benefit compared with two lifetime screens at ages 30 and 45 when the screening ages considered were 25, 45 and 65; however, screens at ages 30, 40 and 55 prevented an additional 2.8% (95% CR: 1.7–3.9) of cancers compared with screening at 30 and 45 only. Compared to the scenario with three lifetime screens (at the best of the ages considered here – ages 30, 40 and 55), having a fourth lifetime screen (at ages 30, 40, 50 and 65) prevented only 1.0% (95% CR: −0.3–2.5) of cancers per additional screen. The incremental benefit of further screens beyond four was below 1%. These results were very similar for the other 16/18 vaccine scenarios considered, with slightly smaller benefits of screening when cross protection was assumed, and slightly larger benefits when the vaccine efficacy was allowed to wane (Table 4). With full screening coverage, the benefits per additional screen are very similar, with an incremental benefit of 2.7% (95% CR: 1.9–3.5) per additional lifetime screen with screening invitations at ages 30, 40 and 55 compared to two screening invites per lifetime at ages 30 and 45, and an incremental benefit of 1.0% (95% CR: 0.4–1.6) per additional screen for four lifetime invites at ages 30, 40, 50 and 65 compared to three at ages 30, 40 and 55.

**Table 4:** Reduction in cancer incidence and incremental benefit (the reduction in cancer incidence per additional screen) for realistic screening coverage, from 10,000 simulated datasets of 300,000 women with natural history parameters drawn from the distributions given in Figure 1, under vaccine I (prevents all HPV‐16/18), vaccine II (prevents all HPV‐16/18 with 15% cross‐protection against other high risk HPV strains), vaccine III (prevents all HPV‐16/18 with 22% cross‐protection against other high risk HPV strains) and vaccine IV (initially prevents all HPV‐16/18, but efficacy wanes by 0.25% every 6 months)

	Vaccine							
	I		II		III		IV	
Screening scenario	% reduction (95% CR)	% incremental benefit per screen (95% CR)	% reduction (95% CR)	% incremental benefit per screen (95% CR)	% reduction (95% CR)	% incremental benefit per screen (95% CR)	% reduction (95% CR)	% incremental benefit per screen (95% CR)
2 screens:								
30, 45	86.0 (83.2, 88.6)	3.9 (2.6, 5.2)	88.1(85.6, 90.5)	3.5 (2.3, 4.8)	88.9 (86.7, 91.0)	3.1 (1.9, 4.3)	83.5(80.4, 86.2)	4.3 (3.0, 5.7)
30, 55	85.5 (82.6, 88.3)	2.8 (1.7, 4.0)	87.7 (85.1, 90.1)	2.5 (1.5, 3.6)	88.6 (86.3, 90.7)	2.3 (1.3, 3.3)	83.2 (80.1, 86.1)	3.4 (2.2, 4.7)
3 screens:								
30, 40, 55	88.3 (85.9, 90.6)	2.8 (1.7, 3.9)	90.0 (87.8, 92.1)	2.2 (1.3, 3.3)	90.8 (89.0, 92.6)	2.3 (1.3, 3.2)	86.3 (83.6, 88.7)	3.3 (2.2, 4.5)
30, 45, 60	87.6 (85.1, 90.0)	2.2 (1.0, 3.4)	89.4 (87.1, 91.5)	1.8 (0.7, 2.9)	90.2 (88.3, 92.1)	1.8 (0.8, 2.9)	85.6 (82.8, 88.0)	2.8 (1.6, 4.1)
25, 45, 65	85.7 (82.8, 88.4)	−0.4 (−1.9, 1.0)	87.7 (85.2, 90.2)	−0.5 (−1.8, 0.8)	88.6 (86.4, 90.8)	−0.5 (−1.8, 0.8)	83.3 (80.3, 86.0)	−0.3 (−1.8, 1.2)
4 screens:								
30, 40, 50, 65	88.9 (86.6, 91.1)	1.0 (−0.3, 2.5)	90.5 (88.4, 92.4)	0.8 (−0.5, 2.2)	91.2 (89.4, 92.9)	0.8 (−0.5, 2.1)	87.0 (84.5, 89.3)	1.3 (−0.3, 2.8)
25, 35, 50, 65	88.6 (86.1, 90.8)	0.4 (−0.9, 1.6)	90.3 (88.2, 92.2)	0.5 (−0.7, 1.7)	91.0 (89.2, 92.7)	0.3 (−0.9, 1.4)	86.7 (84.2, 89.0)	0.6 (−0.7, 2.0)

When considering the nonavalent vaccine, with 100% screening coverage the incremental benefit with three lifetime screens was 1.0% (95% CR: 0.6–1.5) per additional lifetime screen, 1.6% (95% CR: 1.0–2.1) per additional lifetime screen for two lifetime screens, and 5.8% (95% CR: 4.6–7.1) for one lifetime screen compared to no screening. The results were similar with realistic screening coverage. When the analyses were restricted to simulated natural history datasets which produced the highest 2.5% of cumulative risks of cervical cancer up to age 80 (cumulative risk up to age 80 in the absence of vaccination or screening ≥2.7%) and lowest 2.5% of cumulative risks up to age 80 (cumulative risk up to age 80 ≤1.7%), very similar results were found (Supporting Information, Tables S5 and S6).

The results using fixed natural history transition parameters produced very similar point estimates and narrower central ranges for the proportion of cancers prevented and incremental benefit of each additional screen for each vaccine and screening combination, for both full screening coverage and realistic screening coverage (Supporting Information, Tables S3 and S4).

## Discussion

The natural history of cervical cancer is relatively well understood, which makes modelling an attractive option to estimate the impact of changes to screening programmes prior to implementation. We carried out a simulation study to consider the impact of a variety of screening algorithms on the incidence of cervical cancer in unvaccinated women and under four scenarios with varying vaccination efficacy. We consider vaccinated and unvaccinated women separately, as the population will contain a mix of these women, and the appropriate intensity of cervical screening differs between these groups. Our results confirm that even in unvaccinated women, screening intervals can be safely lengthened with the introduction of HPV testing with cytology triage, compared to cytology testing. We benchmarked the incremental benefits of cytology and HPV screening in unvaccinated women to establish boundaries for which screening scenarios to support based on their incremental benefits. In women vaccinated with a 16/18‐vaccine, there was substantial (>2%) incremental benefit of offering three rather than two lifetime screens, whereas the incremental benefit beyond three was limited (0.8–1.3%). These results were consistent for the wide range of transition probabilities and three 16/18‐vaccine scenarios considered. In women vaccinated with the nonavalent vaccine, the incremental benefit beyond two invites was minimal (≤1.1%) and the incremental benefit of two screens over one was small (1.0–1.6%).

By considering realistic screening coverage and perfect screening compliance, and ensuring the natural history model reflects observed HPV prevalence (e.g., in England), we aim to ground the analyses in real world settings, as recommended by Kim *et al*.^5^ We have considered a variety of vaccination scenarios, including a nonavalent vaccine, which is produced by the same company as the quadrivalent vaccine, and is therefore likely to replace the quadrivalent vaccine in the coming years.

As with all simulations, the underlying model is based on numerous assumptions. Natural history models for cervical cancer are well studied; however, advanced precancerous disease is usually treated; therefore there is little evidence on which to base transition probabilities from high‐grade CIN or asymptomatic cancer, and the evidence that does exist mainly comes from one study^18^ in which treatment of CIN3 was withheld in 1965–1974. Lesions that are currently considered CIN3 may differ to a lesion labelled CIN3 50 years ago, and progression rates may vary by age; additionally previous research has shown that progression from CIN3 to cancer in young women cannot occur at the rate suggested by this study.^19^ However, similar conclusions were obtained when the natural history transition probabilities were drawn from distributions reflecting their uncertainty. Our natural history model is relatively simple, excluding hysterectomy and death prior to age 80, though as our outcome is the proportion of cancers prevented, these exclusions cancel out as they are applied to both the numerator and denominator, and should therefore not have a large impact on the results. Additionally, our model is fast to simulate (around 86 sec per simulation, including all 6 vaccine scenarios and 13 screening scenarios, when run on a Linux IBM System X iDataPlex dx360 M3 Server node using a Core Intel Xeon E5645 (Westmere) processor), features far fewer parameters than a more complicated model and is calibrated to observed data in England. Additionally, by selecting parameter values at random from a multivariate normal distribution, we have shown that our results are robust to the choice of parameters and the target population.

Although we have boundaries for which screening scenarios to support based on incremental benefits, accepting screening scenarios with an incremental benefit ≥2.0% and rejecting those ≤0.9%, we provide no guidance as to what to do when the incremental benefit falls between these boundaries. Considering additional scenarios could help narrow this interval, but it is unlikely that this method could be used to define an exact cut‐off. Rather we think that other country‐specific factors should be considered, including the underlying risk of cervical cancer, the GDP, and the existing screening infrastructure.

In previous work, we estimated that, compared to no screening, 74.1% of cervical cancers could be prevented if all women were regularly screened.^20^ This is lower than the proportion estimated in these simulations (86.5%). The simulation with full screening coverage also assumes full compliance to follow‐up appointments, such as repeat screening and colposcopy, whereas our previous estimate only assumed that all women regularly attended the screening appointment, not follow‐up. In addition, Landy *et al*. used empirical data from prior to 2012, when HPV triage was introduced in England, whereas this simulation models cytology with HPV triage, which would increase the estimated proportion of cancers which would be prevented if all women were screened regularly. The estimate with realistic screening coverage, which does not assume full compliance to follow‐up, was 64.3% of cancers prevented compared to no screening in the simulation, and current screening was estimated to prevent 60.5% in the previous research.

We have not incorporated individual‐level information about the simulated women (beyond age), though have allowed 10% of women to be at higher risk of contracting both HPV16/18 and other high‐risk HPV. We do not consider herd immunity, which would be expected to lower the incremental benefit of additional screens in unvaccinated women. We consider only a small range of possible screening scenarios, and a single level of realistic screening coverage. We have only considered women who were fully vaccinated at age 12, prior to HPV exposure, or not vaccinated at all, not women who were partially vaccinated. Despite these limitations, it is reassuring that different models developed by groups working independently produce similar results.

New screening guidelines in the Netherlands, implemented from January 2017, recommend HPV testing at ages 30, 35, 40, 45 and 50 (five lifetime screens) for women whose previous tests were negative,^21^ and Australian guidelines, due to be implemented in December 2017, recommend 5‐yearly screening for women aged 25–74 (10 lifetime screens) using primary HPV testing.^22^ Assuming similar underlying risks and test sensitivities, these simulation results suggest that fewer lifetime screens in these countries would result in a similar incidence rate in vaccinated women. Conversely offering only five lifetime screens to unvaccinated women would lead to an increase in cancer incidence.

Our results are consistent with results from previous simulations studies, which demonstrate that less frequent screening is needed in women vaccinated with a 16/18‐vaccine than unvaccinated women to provide at least the same protection against cervical cancer.^23^, ^24^, ^25^ Screening in women vaccinated with the nonavalent vaccine has been considered in a simulation study by Kim *et al*.,^23^ where 10‐yearly HPV testing from age 30 ($200,000 per QALY) or 35 ($50,000 or $100,000 per QALY) to 64 were the most cost‐efficient strategies. This corresponds to three or four lifetime screens, compared to our result of two lifetime screens; in comparison, Naber *et al*. concluded that the optimal screening strategy for women vaccinated against HPV‐16/18 was three lifetime screens using primary HPV screening with cytology triage.^25^ The range for cost‐effectiveness considered by Kim *et al*. was $50,000–$200,000 per QALY gained, whereas in the United Kingdom, the National Institute for Health and Care Excellence (NICE) considers an intervention to be potentially cost effective at £20,000–£30,000 ($25,814–$38,721) per QALY gained. Our study does not directly consider cost‐effectiveness, but the incremental benefit of each additional screen does so indirectly, with current screening practice having an incremental benefit of 2.0% compared to 6(10)‐yearly screening in unvaccinated women, giving an approximate cut‐off for what reduction in cancer incidence is considered to be cost‐effective in England. In our model, the nonavalent vaccine prevented 88.4% of cervical cancers across the 10,000 parameter sets considered, compared to 85.3% in Kim *et al*.'s simulations. Additionally, the sensitivity of HPV testing and the sensitivity of cytology to high‐grade precancerous disease was higher in our study, reflecting the high‐quality cytology in England.

We are aware of one simulation study which considers screening combined with the nonavalent vaccine in England,^26^ though it evaluates scenarios at a population level, assuming vaccination status will be unknown, rather than separately for vaccinated and unvaccinated women. Additionally, the outcome considered is cervical cancer death, rather than incidence. That simulation concluded four lifetime screens was most cost‐efficient in England, though the difference in discounted life‐years between two and four lifetime screens was around 0.0006 years (5.3 hr), which would be reduced if the time taken to attend screening was accounted for. Assuming realistic screening coverage in our simulations, the incremental benefit acceptable in England appears to be 2.0% per additional screen, corresponding to the prevention of 43 cancers per 100,000 screens (1 cancer per 2301 screens).

Our results clearly demonstrate that the screening programme should be personalised based on vaccination status. This will require the linkage of vaccination status to the screening programme, a process already recommended in England.^27^ In England, there are few inequalities in routine (as opposed to catch up) HPV vaccine uptake by socioeconomic status,^28^ though there is evidence of poor uptake among certain cultural and ethnic groups. Current US guidelines recommend the same screening intervals for women regardless of vaccination status,^4^ though this is likely to be updated once vaccinated women enter screening and the data have been evaluated. Assuming future cervical screening guidelines differ for vaccinated and unvaccinated women, countries which do not have a good record of who received the HPV vaccine may wish to consider the potential use of HPV antibody testing as part of a future cervical screening programme. Whilst herd immunity will lead to a lower risk in unvaccinated women in the vaccination era than prior to vaccination, until the level of herd immunity has been established to be safe to reduce the required frequency of cervical screening, vaccinated and unvaccinated women should have different screening algorithms.

At present, if women in England do not attend screening after an invite, they are sent a reminder, then re‐invited at the age at which they would have been invited had they had a negative test at the missed screen. With longer screening intervals or a small number of lifetime screens, it would be important to re‐invite women at shorter intervals if they fail to attend – the longer intervals modelled in this article are only safe following a negative HPV test.

Our analyses clearly demonstrate that many fewer lifetime screens are necessary for vaccinated women to have the same level of protection against cervical cancer as is currently provided by 3‐ and 5‐yearly cytology screening in unvaccinated women. Different screening algorithms will be appropriate for vaccinated and unvaccinated women, at least until herd immunity data confirms it is safe to reduce screening in unvaccinated women. This emphasizes the importance of recording vaccination status, and linking this information to the screening programmes call–recall database.

## Author Contributions

PDS conceived and designed the work. PW and MSG programmed the microsimulation model. RL designed the microsimulation model, interpreted the results and prepared the first draft of the manuscript. All authors had full access to all the data in the study and confirm that it is an honest, accurate and transparent account of the study being reported; that no important aspects of the study have been omitted and that any discrepancies are disclosed. All authors edited the report and approved the final version. RL had final responsibility for the decision to submit for publication.

## Declaration of Interests

All authors declare no conflicts of interest.

## Supporting information

Supporting Information

Supporting Information

Supporting Information Tables
