# Supplementary material for: What cervical screening is appropriate for women who have been vaccinated against high risk HPV? A simulation study
Source: Int J Cancer. 2017 Nov 10;142(4):709–18. doi: 10.1002/ijc.31094 (PMC5765470; doi:10.1002/ijc.31094)
Supplement: Supplementary file 1 — Supporting Information [file IJC-142-709-s001.docx]

**Supplementary material 1: Assumptions in the microsimulation model**

**Screening assumptions**

**Realistic screening coverage**

For the realistic screening coverage scenario, we used data from the Health and Social Care Information Centre (HSCIC) cervical screening statistical bulletin 2015 ((1), Data tables - Table 3) to determine the proportion of women who attended on time, slightly late, very late and never. For women aged 25-49 at the time of a routine outcome, 71.3% attend on time at 3 years, 12.4% attend at a uniform distribution from 3.5-10 years after a routine outcome, 1.7% attend uniformly between 10-15 years following a routine outcome, and 1.1% attend uniformly 15-20 years after a routine outcome. For women aged 50-64 at the time of a routine outcome, 76.5% attend on time at 5 years, 11.4% attend uniformly between 5-10 years, 3.9% attend uniformly between 10-15 years, and 4.5% attend uniformly between 15-10 years following a routine outcome. Following an early recall, 85.1% return on time, 8.9% return at a uniform distribution over the following year, and the remaining 6.0% return at an exponential distribution, a mean of 3 years late, with a variance of four years.

**Screening algorithms and test sensitivity**

At each routine screening event an HPV test with 96% sensitivity to HPV was performed (2); for those who tested HPV positive a cytology test was carried out, with 70% sensitivity for low-grade CIN, 85% for high-grade CIN and 92% for asymptomatic cancer.

If women with high grade CIN have a positive cytology triage test, then 90% are assumed to be treated ‘successfully’ (allowing for some treatment failure and some women not to return for treatment). 5% of low-grade CIN are also assumed to be treated ‘successfully’ (i.e. preventing possible future progression to high grade CIN without further infection). Women who are HPV positive but cytology negative are recalled one year later, and if still HPV positive and cytology negative are recalled once more one year later. After three consecutive HPV-positive and cytology-negative results, women are referred to colposcopy, where 90% of high-grade CIN is treated ‘successfully’, and women with HPV or low-grade CIN are invited back after 2 years if they have HPV16/18 or 3 years if they have another high-risk HPV infection. Once ‘successfully’ treated, women who would have developed cancer in the absence of screening have a 0.09% per annum chance of developing cancer (3).

For scenarios A’ and B’, with primary cytology screening and HPV triage, women are referred to colposcopy if both cytology and HPV tests are positive; otherwise they have the routine screening interval of 3 or 5 years under A’ and 6 or 10 years under B’. Among all women, we set the sensitivity of cytology to low-grade CIN to be 63.5%, to high-grade CIN to 72.5%, and to asymptomatic cancer to 92%. The sensitivity to high-grade CIN is slightly lower than that reported in the HART study (4), to reflect that cytology in trials is normally slightly better than in a population based programme.

**Vaccination assumptions**

The level of protection provided by the quadrivalent vaccine against persistent specific HPV-strains was taken from Brown et al (5), and the distribution of HPV-strains in cancers was taken from Clifford et al (6). For protection against persistent HPV infection for the bivalent vaccine, data from the PATRICIA study was used, as much heterogeneity was found in a meta-analysis (7), implying the results should not be combined. These were used to calculate the proportion of cancers are not caused by HPV-16/18 which are caused by each of the other HPV strains partially prevented by the quadrivalent and bivalent vaccines. Multiplying the proportion of these HPV infections which would be prevented by the proportion of non-16/18 cancers caused by each HPV infection gives the proportion of non-16/18 cancers which would be prevented by the cross-protection provided against that HPV strain, and summing these gives the total cross-protection against cancer provided by the quadrivalent vaccine as 14.7%, and the bivalent vaccine as 22.1%.

| HPV type | % of cancers caused by HPV strain (1) | % of non-HPV-16/18 cancers | protection by quadrivalent vaccine (2) | % of non16/18cancers prevented through cross-protection provided by the quadrivalent vaccine | protection by bivalent vaccine (3) | % of non16/18cancers prevented through cross-protection provided by the quadrivalent vaccine |
| --- | --- | --- | --- | --- | --- | --- |
| 16 | 54.6 |  |  |  |  |  |
| 18 | 15.8 |  |  |  |  |  |
| 45 | 3.7 | 12.5 | 0.078 | 1.0 | 0.736 | 9.2 |
| 31 | 3.5 | 11.8 | 0.462 | 5.5 | 0.768 | 9.1 |
| 33 | 4.4 | 14.9 | 0.287 | 4.3 | 0.448 | 6.7 |
| 58 | 3.4 | 11.5 | 0.055 | 0.6 | -0.183 | -2.1 |
| 52 | 2.5 | 8.4 | 0.184 | 1.6 | 0.083 | 0.7 |
| 35 | 1.8 | 6.1 | 0.178 | 1.1 | -0.198 | -1.2 |
| 59 | 1.1 | 3.7 | 0.187 | 0.7 | -0.075 | -0.3 |
| 56 | 0.8 | 2.7 | n/a |  | -0.053 | -0.1 |
| 51 | 0.7 | 2.4 | n/a |  | 0.166 | 0.4 |
| 68 | 0.5 | 1.7 | n/a |  | 0.026 | 0.0 |
| 39 | 0.7 | 2.4 | n/a |  | 0.048 | 0.1 |
| % of all cervical cancers not caused by HPV-16/18 | 29.6 |  |  | 14.7 |  | 22.1 |

References

1. Screening and Immunisations Team Health and Social Care Information Centre. Cervical Screening Programme, England - Statistics for 2014-15. 2015.

2. Arbyn M, Ronco G, Anttila A, Meijer CJ, Poljak M, Ogilvie G, Koliopoulos G, Naucler P, Sankaranarayanan R, Peto J. Evidence regarding human papillomavirus testing in secondary prevention of cervical cancer. Vaccine. 2012;30:F88-F99.

3. Soutter W, de Barros Lopes A, Fletcher A, Monaghan J, Duncan I, Paraskevaidis E, Kitchener H. Invasive cervical cancer after conservative therapy for cervical intraepithelial neoplasia. The Lancet. 1997;349(9057):978-80.

4. Cuzick J, Szarewski A, Cubie H, Hulman G, Kitchener H, Luesley D, McGoogan E, Menon U, Terry G, Edwards R. Management of women who test positive for high-risk types of human papillomavirus: the HART study. The Lancet. 2003;362(9399):1871-6.

5. Brown DR, Kjaer SK, Sigurdsson K, Iversen O-E, Hernandez-Avila M, Wheeler CM, Perez G, Koutsky LA, Tay EH, Garcia P. The impact of quadrivalent human papillomavirus (HPV; types 6, 11, 16, and 18) L1 virus-like particle vaccine on infection and disease due to oncogenic nonvaccine HPV types in generally HPV-naive women aged 16–26 years. J Infect Dis. 2009;199(7):926-35.

6. Clifford G, Franceschi S, Diaz M, Munoz N, Villa LL. HPV type-distribution in women with and without cervical neoplastic diseases. Vaccine. 2006;24:S26-S34.

7. Malagón T, Drolet M, Boily M-C, Franco EL, Jit M, Brisson J, Brisson M. Cross-protective efficacy of two human papillomavirus vaccines: a systematic review and meta-analysis. The Lancet infectious diseases. 2012;12(10):781-9.
