# Supplementary material for: What cervical screening is appropriate for women who have been vaccinated against high risk HPV? A simulation study
Source: Int J Cancer. 2017 Nov 10;142(4):709–18. doi: 10.1002/ijc.31094 (PMC5765470; doi:10.1002/ijc.31094)
Supplement: Supplementary file 2 — Supporting Information [file IJC-142-709-s002.docx]

**Titles and legends to supporting** **information**:

Supplementary material 1: Assumptions in the microsimulation model

Table S1: Number of screens, cancers, reduction in cancer incidence and incremental benefit (the reduction in cancer incidence per additional screen) for 100% screening coverage, for each combination of vaccination and screening scenario, from 10,000 simulated datasets of 300,000 women with natural history parameters drawn from the distributions given in Appendix Table 1

Table S2: Number of screens, cancers, reduction in cancer incidence and incremental benefit (the reduction in cancer incidence per additional screen) for realistic screening coverage, for each combination of vaccination and screening scenario, from 10,000 simulated datasets of 300,000 women with natural history parameters drawn from the distributions given in Appendix Table 1

Table S3: Number of screens, cancers, reduction in cancer incidence and incremental benefit (the reduction in cancer incidence per additional screen) for 100% screening coverage, for each combination of vaccination and screening scenario, from 1,000 simulated datasets of 300,000 women with fixed natural history parameters given in Appendix 1

Table S4: Number of screens, cancers, reduction in cancer incidence and incremental benefit (the reduction in cancer incidence per additional screen) for realistic screening coverage, for each combination of vaccination and screening scenario, from 1,000 simulated datasets of 300,000 women with fixed natural history parameters given in Appendix 1

Table S5: Number of screens, cancers, reduction in cancer incidence and incremental benefit (the reduction in cancer incidence per additional screen) for 100% screening coverage, for each combination of vaccination and screening scenario, from simulated datasets of 300,000 women with natural history parameters drawn from the distributions given in Appendix Table 1 which had a lifetime risk of cervical cancer in the absence of vaccination or screening ≤1.7%

Table S6: Number of screens, cancers, reduction in cancer incidence and incremental benefit (the reduction in cancer incidence per additional screen) for 100% screening coverage, for each combination of vaccination and screening scenario, from simulated datasets of 300,000 women with natural history parameters drawn from the distributions given in Appendix Table 1 which had a lifetime risk of cervical cancer in the absence of vaccination or screening ≥2.7%
