# Supplementary material for: What cervical screening is appropriate for women who have been vaccinated against high risk HPV? A simulation study
Source: Int J Cancer. 2017 Nov 10;142(4):709–18. doi: 10.1002/ijc.31094 (PMC5765470; doi:10.1002/ijc.31094)
Supplement: Supplementary file 3 — Supporting Information Tables [file IJC-142-709-s003.pdf]

Table S1: Number of screens, cancers, reduction in cancer incidence and incremental benefit (the reduction in cancer incidence per additional screen) for 100% screening coverage, for each combination of vaccination and screening scenario, from 10,000 simulated datasets of 300,000 women with natural history parameters drawn from the distributions given in Figure 1

Vaccine	Screening scenario*	Number of screens	Number of cancers (per cohort of 300,000)		Cancers prevented				Incremental benefit	
		Mean (sd)	Mean	95% CR	%	95% CR	Minimum	Maximum	%	95% CR
None	None		6576	(5002, 8060)						
	HPV primary testing									
	3/5-yearly	12.7 (0.0)	592	(481, 703)	90.9	(88.9, 92.5)	86.9	93.7	0.8	(0.6, 1.0)
	6/10-yearly	7.5 (0.0)	868	(713, 1018)	86.7	(84.0, 88.8)	81.0	90.6	2.6	(2.2, 3.1)
	30, 40, 50, 65	4.4 (0.0)	1411	(1149, 1654)	78.4	(74.8, 81.1)	71.0	83.5	3.0	(2.0, 4.1)
	30, 40, 55	3.4 (0.0)	1605	(1305, 1877)	75.5	(71.5, 78.5)	66.9	81.2	22.2	(21.0, 23.3)
	Cytology primary testing									
	3/5-yearly	12.0 (0.0)	864	(709, 1016)	86.5	(83.8, 88.7)	81.2	90.1	1.6	(1.3, 1.8)
	6/10-yearly	7.0 (0.0)	1369	(1126, 1595)	78.7	(75.2, 81.6)	70.5	84.2	11.2	(10.7, 11.7)
I	None		1949	(1419, 2526)	70.3	(65.1, 75.5)	62.7	77.6		
	3/5-yearly	12.3 (0.0)	195	(145, 252)	97.0	(96.0, 97.8)	95.2	98.2	0.3	(0.2, 0.4)
	6/10-yearly	7.3 (0.0)	280	(212, 358)	95.7	(94.4, 96.8)	93.4	97.5	0.8	(0.5, 1.0)
	30, 40, 50, 65	4.2 (0.0)	433	(330, 549)	93.4	(91.6, 94.8)	90.3	95.7	1.0	(0.4, 1.6)
	25, 35, 50, 65	4.3 (0.0)	467	(353, 591)	92.8	(90.9, 94.5)	89.9	95.6	0.4	(-0.2, 1.1)
	30, 40, 55	3.2 (0.0)	499	(377, 634)	92.4	(90.5, 94.0)	89.0	95.0	2.7	(1.9, 3.5)
	30, 45, 60	3.2 (0.0)	557	(422, 707)	91.5	(89.4, 93.3)	88.1	94.5	1.9	(1.2, 2.6)
	25, 45, 65	3.2 (0.0)	704	(532, 896)	89.2	(86.7, 91.4)	85.0	92.6	-0.4	(-1.2, 0.4)
	30, 45	2.2 (0.0)	682	(510, 868)	89.6	(87.3, 91.7)	85.6	92.8	3.9	(2.9, 5.0)
	30, 55	2.2 (0.0)	752	(565, 959)	88.5	(85.9, 90.9)	84.4	92.3	2.9	(1.9, 4.0)
	35	1.1 (0.0)	959	(718, 1221)	85.4	(82.3, 88.2)	80.1	89.6	14.0	(11.3, 16.8)
	40	1.1 (0.0)	1005	(756, 1272)	84.7	(81.5, 87.5)	78.8	89.1	13.6	(10.8, 16.4)
	30	1.1 (0.0)	1019	(753, 1313)	84.4	(81.3, 87.4)	79.2	89.2	12.4	(10.0, 15.0)
	45	1.0 (0.0)	1168	(878, 1478)	82.2	(78.7, 85.5)	76.5	87.2	11.5	(9.1, 14.1)
	None		1706	(1232, 2217)	74.0	(69.5, 78.6)	67.3	80.4		
	3/5-yearly	12.3 (0.0)	157	(116, 203)	97.6	(96.9, 98.2)	95.7	98.5	0.2	(0.1, 0.3)
	6/10-yearly	7.2 (0.0)	228	(169, 293)	96.5	(95.5, 97.3)	94.3	97.9	0.7	(0.5, 1.0)
	30, 40, 50, 65	4.2 (0.0)	375	(283, 476)	94.3	(92.8, 95.6)	91.0	96.5	0.8	(0.3, 1.4)

II	25, 35, 50, 65	4.3 (0.0)	387	(290, 494)	94.1	(92.5, 95.5)	91.0	96.3	0.6	(0.0, 1.2)
	30, 40, 55	3.2 (0.0)	428	(321, 547)	93.4	(91.8, 95.0)	90.5	95.9	2.1	(1.4, 2.9)
	30, 45, 60	3.1 (0.0)	472	(352, 606)	92.8	(90.9, 94.4)	89.4	95.6	1.5	(0.9, 2.2)
	25, 45, 65	3.1 (0.0)	608	(450, 781)	90.7	(88.6, 92.7)	86.9	93.8	-0.5	(-1.2, 0.2)
	30, 45	2.1 (0.0)	572	(427, 732)	91.3	(89.1, 93.2)	87.2	94.2	3.5	(2.6, 4.5)
	30, 55	2.1 (0.0)	634	(477, 816)	90.3	(88.1, 92.4)	86.1	93.5	2.6	(1.7, 3.6)
	35	1.1 (0.0)	818	(612, 1047)	87.5	(84.9, 90.1)	83.0	91.5	12.7	(10.1, 15.2)
	40	1.0 (0.0)	873	(654, 1109)	86.7	(83.8, 89.5)	82.2	91.0	12.1	(9.6, 14.6)
	30	1.1 (0.0)	865	(642, 1117)	86.8	(84.0, 89.5)	81.9	91.2	11.4	(9.1, 13.7)
	45	1.0 (0.0)	1018	(749, 1302)	84.5	(81.3, 87.6)	79.6	89.3	10.2	(8.1, 12.4)
III	None		1552	(1124, 2022)	76.4	(72.2, 80.6)	70.3	82.1		
	3/5-yearly	12.3 (0.0)	153	(111, 198)	97.7	(97.1, 98.2)	96.3	98.6	0.2	(0.1, 0.3)
	6/10-yearly	7.2 (0.0)	220	(163, 281)	96.6	(95.8, 97.4)	95.1	97.8	0.6	(0.4, 0.8)
	30, 40, 50, 65	4.1 (0.0)	346	(261, 437)	94.7	(93.4, 95.8)	92.5	96.4	0.8	(0.3, 1.3)
	25, 35, 50, 65	4.2 (0.0)	366	(271, 468)	94.4	(93.2, 95.6)	92.3	96.3	0.4	(-0.1, 1.0)
	30, 40, 55	3.2 (0.0)	395	(299, 500)	94.0	(92.5, 95.2)	91.4	96.0	2.2	(1.5, 2.9)
	30, 45, 60	3.1 (0.0)	438	(330, 556)	93.3	(91.8, 94.7)	90.6	95.4	1.6	(1.0, 2.3)
	25, 45, 65	3.1 (0.0)	571	(423, 732)	91.3	(89.4, 93.1)	88.2	94.0	-0.4	(-1.2, 0.3)
	30, 45	2.1 (0.0)	544	(407, 692)	91.7	(89.9, 93.4)	88.5	94.2	3.1	(2.2, 4.0)
	30, 55	2.1 (0.0)	597	(446, 761)	90.9	(89.0, 92.7)	87.6	93.6	2.3	(1.5, 3.2)
	35	1.1 (0.0)	761	(558, 980)	88.4	(86.1, 90.6)	84.8	91.7	11.4	(9.1, 13.6)
	40	1.0 (0.0)	790	(589, 1011)	87.9	(85.6, 90.2)	83.9	91.5	11.1	(8.9, 13.4)
	30	1.1 (0.0)	815	(597, 1048)	87.6	(85.2, 89.9)	83.6	91.1	10.1	(8.1, 12.2)
	45	1.0 (0.0)	926	(692, 1179)	85.9	(83.1, 88.5)	81.2	90.1	9.3	(7.3, 11.3)
IV	None		2244	(1690, 2846)	65.8	(60.8, 70.8)	57.9	73.2		
	3/5-yearly	12.3 (0.0)	229	(179, 284)	96.5	(95.4, 97.3)	94.5	97.9	0.3	(0.2, 0.4)
	6/10-yearly	7.3 (0.0)	325	(255, 400)	95.0	(93.6, 96.2)	92.4	97.0	0.9	(0.7, 1.2)
	30, 40, 50, 65	4.2 (0.0)	514	(407, 632)	92.1	(90.1, 93.9)	88.7	95.0	1.3	(0.7, 2.0)
	25, 35, 50, 65	4.3 (0.0)	538	(423, 664)	91.8	(89.7, 93.4)	88.3	94.6	0.8	(0.2, 1.5)
	30, 40, 55	3.2 (0.0)	599	(472, 737)	90.8	(88.6, 92.8)	86.8	93.9	3.2	(2.4, 4.0)
	30, 45, 60	3.2 (0.0)	649	(508, 796)	90.1	(87.8, 92.1)	86.1	93.2	2.5	(1.8, 3.4)
	25, 45, 65	3.2 (0.0)	830	(648, 1023)	87.3	(84.6, 89.7)	82.6	90.9	-0.2	(-1.0, 0.6)
	30, 45	2.2 (0.0)	817	(645, 1002)	87.5	(84.8, 89.8)	82.6	91.1	4.3	(3.3, 5.4)

	30, 55	2.2 (0.0)	876	(687, 1085)	86.6	(83.7, 89.1)	81.5	90.5	3.5	(2.5, 4.6))
	35	1.1 (0.0)	1128	(871, 1399)	82.8	(79.5, 85.7)	77.4	87.3	15.8	(13.1, 18.4)
	40	1.1 (0.0)	1185	(923, 1454)	81.9	(78.5, 85.0)	75.9	86.7	15.2	(12.5, 17.9)
	30	1.2 (0.0)	1204	(926, 1503)	81.6	(78.2, 84.7)	75.9	86.6	13.7	(11.4, 16.2)
	45	1.0 (0.0)	1366	(1065, 1674)	79.1	(75.3, 82.6)	72.7	84.6	12.9	(10.5, 15.4)
V	None		765	(556, 1017)	88.4	(86.1, 90.5)	85.1	91.9		
	3/5-yearly	12.1 (0.0)	78	(53, 106)	98.8	(98.3, 99.2)	97.9	99.5	0.1	(0.0, 0.2)
	6/10-yearly	7.1 (0.0)	112	(80, 150)	98.3	(97.6, 98.8)	97.2	99.1	0.3	(0.2, 0.5)
	30, 40, 50, 65	4.1 (0.0)	176	(129, 229)	97.3	(96.5, 98.0)	95.7	98.4	0.4	(0.0, 0.7)
	25, 35, 50, 65	4.1 (0.0)	183	(133, 243)	97.2	(96.3, 97.9)	95.4	98.4	0.2	(-0.1, 1.0)
	30, 40, 55	3.1 (0.0)	199	(147, 259)	97.0	(96.1, 97.7)	95.3	98.2	1.0	(0.6, 1.5)
	30, 45, 60	3.1 (0.0)	221	(163, 288)	96.6	(95.7, 97.4)	95.0	98.0	0.7	(0.3, 1.1)
	25, 45, 65	3.1 (0.0)	276	(200, 365)	95.8	(94.6, 96.8)	93.6	97.4	-0.1	(-0.6, 0.3)
	30, 45	2.1 (0.0)	268	(198, 349)	95.9	(94.8, 96.9)	94.1	97.4	1.6	(1.0, 2.1)
	30, 55	2.1 (0.0)	298	(221, 389)	95.4	(94.3, 96.5)	93.4	97.1	1.1	(0.6, 1.7)
	35	1.0 (0.0)	373	(276, 488)	94.3	(93.0, 95.5)	92.1	96.2	5.8	(4.6, 7.1)
	40	1.0 (0.0)	390	(291, 505)	94.0	(92.6, 95.3)	91.4	96.0	5.6	(4.3, 6.9)
	30	1.1 (0.0)	397	(290, 526)	94.0	(92.6, 95.2)	91.6	95.9	5.3	(4.2, 6.5)
	45	1.0 (0.0)	459	(341, 597)	93.0	(91.3, 94.5)	90.3	95.4	4.6	(3.5, 5.7)

Central Range, the 2.5th and 97.5th percentiles

Vaccine I: 100% efficacy against HPV16/18, no cross protection or waning efficacy

Vaccine II: 100% efficacy against HPV16/18, cross protection for the quadrivalent vaccine, preventing 14.7% of other high risk HPV infections

Vaccine III: 100% efficacy against HPV16/18, cross protection for the bivalent vaccine, preventing 22.1% of other high risk HPV infections

Vaccine IV: 100% initial efficacy against HPV16/18, but efficacy wanes by 0.25% (absolute) every 6 months

Vaccine V: the nonavalent vaccine, providing 100% efficacy against HPV16/18 and preventing 63.6% of other high risk HPV infections

*Screening scenarios provide screening ages, except 3/5 (6/10)-yearly represents screening every 3(6) years aged 25-49 years and every 5(10) years aged 50-64 years.

Table S2: Number of screens, cancers, reduction in cancer incidence and incremental benefit (the reduction in cancer incidence per additional screen) for realistic screening coverage, for each combination of vaccination and screening scenario, from 10,000 simulated datasets of 300,000 women with natural history parameters drawn from the distributions given in Figure 1

Vaccine	Screening scenario*	Number of screens	Number of cancers (per cohort of 300,000)		Cancers prevented				Incremental benefit	
		Mean (sd)	Mean	95% CR	%	95% CR	Minimum	Maximum	%	95% CR
None	None		6576	(5002, 8060)						
	HPV primary testing									
	3/5-yearly	7.2 (0.0)	1885	(1501, 2241)	71.2	(68.7, 73.2)	66.0	74.9	0.9	(0.3, 1.7)
	6/10-yearly	5.1 (0.0)	2013	(1604, 2387)	69.3	(66.5, 71.5)	63.7	72.9	3.0	(2.2, 3.8)
	30, 40, 50, 65	3.2 (0.0)	2375	(1897, 2813)	63.8	(60.4, 66.4)	57.4	69.2	3.1	(0.5, 5.8)
	30, 40, 55	2.7 (0.0)	2484	(1987, 2941)	62.1	(58.6, 64.9)	54.5	67.4	23.1	(21.8, 24.3)
	Cytology primary testing									
	3/5-yearly	6.6 (0.0)	2298	(1842, 2727)	64.3	(61.3, 66.8)	56.9	68.4	2.0	(1.2, 2.8)
	6/10-yearly	4.6 (0.0)	2554	(2043, 3026)	60.3	(57.0, 63.1)	53.5	65.4	13.1	(12.4, 13.7)
I	None		1949	(1419, 2526)	70.3	(65.1, 75.5)	62.7	77.6		
	3/5-yearly	6.9 (0.0)	591	(441, 752)	91.0	(89.1, 92.7)	87.9	93.7	0.3	(-0.1, 0.7)
	6/10-yearly	4.8 (0.0)	627	(467, 799)	90.4	(88.4, 92.3)	87.2	93.4	0.9	(0.4, 1.4)
	30, 40, 50, 65	3.1 (0.0)	726	(545, 924)	88.9	(86.6, 91.1)	85.0	92.1	1.0	(-0.3, 2.5)
	25, 35, 50, 65	3.2 (0.0)	748	(559, 950)	88.6	(86.1, 90.8)	84.8	92.1	0.4	(-0.9, 1.6)
	30, 40, 55	2.5 (0.0)	763	(573, 972)	88.3	(85.9, 90.6)	84.3	91.8	2.8	(1.7, 3.9)
	30, 45, 60	2.4 (0.0)	813	(607, 1033)	87.6	(85.1, 90.0)	83.0	91.4	2.2	(1.0, 3.4)
	25, 45, 65	2.3 (0.0)	935	(697, 1196)	85.7	(82.8, 88.4)	81.2	89.6	-0.4	(-1.9, 1.0)
	30, 45	1.7 (0.0)	920	(684, 1173)	86.0	(83.2, 88.6)	81.5	90.2	3.9	(2.6, 5.2)
	30, 55	1.8 (0.0)	948	(709, 1211)	85.5	(82.6, 88.3)	80.8	90.0	2.8	(1.7, 4.0)
	35	0.9 (0.0)	1112	(827, 1419)	83.0	(79.7, 86.1)	77.6	87.4	13.8	(11.1, 16.6)
	40	0.9 (0.0)	1165	(871, 1482)	82.2	(78.8, 85.4)	76.7	87.3	12.6	(10.4, 15.9)
	30	1.0 (0.0)	1147	(848, 1474)	82.5	(79.1, 85.7)	77.3	87.4	13.1	(10.0, 15.2)
	45	0.9 (0.0)	1307	(977, 1658)	80.1	(76.2, 83.7)	74.1	85.6	10.9	(8.6, 13.4)
	None		1706	(1232, 2217)	74.0	(69.5, 78.6)	67.3	80.4		
	3/5-yearly	6.8 (0.0)	499	(368, 640)	92.4	(90.8, 93.9)	89.4	94.8	0.2	(-0.1, 0.6)
	6/10-yearly	4.8 (0.0)	532	(394, 683)	91.9	(90.2, 93.5)	88.9	94.4	0.8	(0.4, 1.3)
	30, 40, 50, 65	3.1 (0.0)	625	(467, 798)	90.5	(88.4, 92.4)	87.1	93.4	0.8	(-0.5, 2.2)

II	25, 35, 50, 65	3.2 (0.0)	636	(473, 816)	90.3	(88.2, 92.2)	86.8	93.3	0.5	(-0.7, 1.7)
	30, 40, 55	2.5 (0.0)	656	(486, 839)	90.0	(87.8, 92.1)	86.6	93.1	2.2	(1.3, 3.3)
	30, 45, 60	2.4 (0.0)	695	(516, 888)	89.4	(87.1, 91.5)	85.8	92.8	1.8	(0.7, 2.9)
	25, 45, 65	2.3 (0.0)	803	(594, 1035)	87.7	(85.2, 90.2)	83.6	91.4	-0.5	(-1.8, 0.8)
	30, 45	1.7 (0.0)	782	(579, 1005)	88.1	(85.6, 90.5)	83.8	91.9	3.5	(2.3, 4.8)
	30, 55	1.8 (0.0)	808	(600, 1039)	87.7	(85.1, 90.1)	83.7	91.4	2.5	(1.5, 3.6)
	35	0.9 (0.0)	954	(712, 1223)	85.4	(82.6, 88.3)	81.0	89.7	12.5	(9.9, 15.0)
	40	0.9 (0.0)	1012	(753, 1290)	84.5	(81.5, 87.5)	80.0	89.3	11.5	(9.3, 14.1)
	30	1.0 (0.0)	980	(727, 1264)	85.0	(82.1, 88.0)	80.4	89.4	11.7	(9.2, 13.8)
	45	0.9 (0.0)	1137	(835, 1457)	82.7	(79.3, 86.0)	77.4	87.7	9.7	(7.7, 11.9)
III	None		1552	(1124, 2022)	76.4	(72.2, 80.6)	70.3	82.1		
	3/5-yearly	6.8 (0.0)	465	(339, 597)	92.9	(91.5, 94.3)	90.8	95.1	0.2	(-0.1, 0.6)
	6/10-yearly	4.8 (0.0)	495	(361, 636)	92.5	(90.9, 93.9)	89.9	94.9	0.7	(0.3, 1.2)
	30, 40, 50, 65	3.1 (0.0)	575	(428, 735)	91.2	(89.4, 92.9)	88.3	93.8	0.8	(-0.5, 2.1)
	25, 35, 50, 65	3.2 (0.0)	592	(436, 760)	91.0	(89.2, 92.7)	88.1	93.6	0.3	(-0.9, 1.4)
	30, 40, 55	2.5 (0.0)	602	(446, 773)	90.8	(89.0, 92.6)	87.5	93.5	2.3	(1.3, 3.2)
	30, 45, 60	2.4 (0.0)	641	(476, 820)	90.2	(88.3, 92.1)	86.9	92.9	1.8	(0.8, 2.9)
	25, 45, 65	2.3 (0.0)	748	(550, 961)	88.6	(86.4, 90.8)	85.0	92.0	-0.5	(-1.8, 0.8)
	30, 45	1.7 (0.0)	729	(540, 933)	88.9	(86.7, 91.0)	85.0	92.1	3.1	(1.9, 4.3)
	30, 55	1.8 (0.0)	750	(555, 961)	88.6	(86.3, 90.7)	85.2	91.9	2.3	(1.3, 3.3)
	35	0.9 (0.0)	880	(646, 1135)	86.6	(84.1, 89.0)	82.5	89.9	11.2	(8.9, 13.5)
	40	0.9 (0.0)	918	(679, 1179)	86.0	(83.4, 88.6)	81.6	89.7	10.2	(8.5, 13.0)
	30	1.0 (0.0)	912	(667, 1179)	86.1	(83.5, 88.7)	81.8	90.2	10.7	(8.2, 12.4)
	45	0.9 (0.0)	1034	(767, 1323)	84.2	(81.2, 87.1)	79.2	88.7	8.9	(7.0, 10.9)
IV	None		2244	(1690, 2846)	65.8	(60.8, 70.8)	57.9	73.2		
	3/5-yearly	6.9 (0.0)	688	(536, 850)	89.5	(87.5, 91.3)	85.8	92.3	0.3	(-0.1, 0.7)
	6/10-yearly	4.9 (0.0)	729	(567, 899)	88.8	(86.7, 90.7)	85.1	92.0	1.0	(0.5, 1.6)
	30, 40, 50, 65	3.1 (0.0)	849	(663, 1040)	87.0	(84.5, 89.3)	82.6	90.9	1.3	(-0.3, 2.8)
	25, 35, 50, 65	3.2 (0.0)	869	(677, 1072)	86.7	(84.2, 89.0)	82.1	90.3	0.6	(-0.7, 2.0)
	30, 40, 55	2.6 (0.0)	895	(698, 1099)	86.3	(83.6, 88.7)	81.9	90.3	3.3	(2.2, 4.5)
	30, 45, 60	2.4 (0.0)	944	(736, 1163)	85.6	(82.8, 88.0)	81.1	89.5	2.8	(1.6, 4.1)
	25, 45, 65	2.3 (0.0)	1093	(849, 1354)	83.3	(80.3, 86.0)	78.2	87.5	-0.3	(-1.8, 1.2)
	30, 45	1.7 (0.0)	1083	(842, 1339)	83.5	(80.4, 86.2)	77.9	87.8	4.3	(3.0, 5.7)

	30, 55	1.8 (0.0)	1098	(849, 1360)	83.2	(80.1, 86.1)	78.4	87.6	3.4	(2.2, 4.7)
	35	0.9 (0.0)	1300	(1001, 1615)	80.1	(76.7, 83.3)	74.4	85.4	15.5	(12.9, 18.1)
	40	0.9 (0.0)	1363	(1055, 1681)	79.2	(75.6, 82.5)	73.4	84.4	13.9	(12.1, 17.3)
	30	1.0 (0.0)	1346	(1032, 1677)	79.5	(75.9, 82.7)	73.3	84.6	14.7	(11.5, 16.4)
	45	0.9 (0.0)	1518	(1177, 1874)	76.8	(72.8, 80.5)	70.3	82.3	12.3	(10.0, 14.8)
V	None		765	(556, 1017)	88.4	(86.1, 90.5)	85.1	91.9		
	3/5-yearly	6.7 (0.0)	230	(167, 302)	96.5	(95.6, 97.3)	95.0	97.9	0.1	(-0.1, 0.4)
	6/10-yearly	4.7 (0.0)	246	(179, 322)	96.2	(95.3, 97.1)	94.6	97.6	0.4	(0.1, 0.7)
	30, 40, 50, 65	3.0 (0.0)	287	(212, 374)	95.6	(94.5, 96.6)	93.8	97.1	0.4	(-0.5, 1.2)
	25, 35, 50, 65	3.1 (0.0)	292	(215, 385)	95.5	(94.4, 96.5)	93.6	97.1	0.2	(-0.6, 1.0)
	30, 40, 55	2.5 (0.0)	300	(222, 390)	95.4	(94.3, 96.4)	93.5	96.9	1.1	(0.5, 1.7)
	30, 45, 60	2.4 (0.0)	319	(235, 418)	95.1	(94.0, 96.2)	93.2	96.8	0.8	(0.1, 1.5)
	25, 45, 65	2.2 (0.0)	365	(268, 481)	94.4	(93.1, 95.6)	92.0	96.3	-0.2	(-1.1, 0.7)
	30, 45	1.6 (0.0)	359	(264, 470)	94.5	(93.3, 95.7)	92.3	96.3	1.5	(0.8, 2.3)
	30, 55	1.7 (0.0)	372	(275, 489)	94.3	(93.0, 95.5)	92.2	96.2	1.1	(0.4, 1.8)
	35	0.9 (0.0)	432	(321, 566)	93.4	(92.0, 94.7)	91.2	95.7	5.7	(4.5, 7.0)
	40	0.9 (0.0)	453	(336, 591)	93.1	(91.6, 94.5)	90.5	95.3	5.3	(4.2, 6.6)
	30	0.9 (0.0)	446	(326, 587)	93.2	(91.7, 94.6)	90.6	95.3	5.4	(4.2, 6.6)
	45	0.9 (0.0)	512	(378, 668)	92.2	(90.4, 93.8)	89.2	94.6	4.4	(3.3, 5.5)

Central Range, the 2.5th and 97.5th percentiles

Vaccine I: 100% efficacy against HPV16/18, no cross protection or waning efficacy

Vaccine II: 100% efficacy against HPV16/18, cross protection for the quadrivalent vaccine, preventing 14.7% of other high risk HPV infections

Vaccine III: 100% efficacy against HPV16/18, cross protection for the bivalent vaccine, preventing 22.1% of other high risk HPV infections

Vaccine IV: 100% initial efficacy against HPV16/18, but efficacy wanes by 0.25% (absolute) every 6 months

Vaccine V: the nonavalent vaccine, providing 100% efficacy against HPV16/18 and preventing 63.6% of other high risk HPV infections

*Screening scenarios provide screening ages, except 3/5 (6/10)-yearly represents screening every 3(6) years aged 25-49 years and every 5(10) years aged 50-64 years.

Table S3: Number of screens, cancers, reduction in cancer incidence and incremental benefit (the reduction in cancer incidence per additional screen) for 100% screening coverage, for each combination of vaccination and screening scenario, from 1,000 simulated datasets of 300,000 women with fixed natural history parameters given in Figure 1

Vaccine	Screening scenario*	Number of screens	Number of cancers (per cohort of 300,000)		Cancers prevented				Incremental benefit	
		Mean (sd)	Mean	95% CR	%	95% CR	Minimum	Maximum	%	95% CR
None	None		6835	(6680, 6994)						
	HPV primary testing									
	3/5-yearly	12.7 (0.0)	608	(559, 658)	91.1	(90.4, 91.8)	89.9	92.1	0.8	(0.7, 1.0)
	6/10-yearly	7.5 (0.0)	896	(837, 959)	86.9	(86.1, 87.7)	85.7	88.1	2.6	(2.3, 2.9)
	30, 40, 50, 65	4.4 (0.0)	1462	(1392, 1538)	78.6	(77.6, 79.5)	76.7	80.6	2.9	(2.0, 3.9)
	30, 40, 55	3.4 (0.0)	1656	(1577, 1737)	75.8	(74.7, 76.8)	74.0	77.4	22.3	(22.0, 22.6)
	Cytology primary testing									
	3/5-yearly	11.9 (0.0)	1221	(1154, 1289)	82.1	(81.2, 83.0)	80.6	83.4	1.3	(1.1, 1.5)
	6/10-yearly	7.0 (0.0)	1661	(1589, 1743)	75.7	(74.7, 76.7)	74.1	77.2	10.9	(10.7, 11.0)
I	None		2050	(1961, 2142)	70.0	(68.5, 71.4)	67.5	72.4		
	3/5-yearly	12.3 (0.0)	196	(171, 225)	97.1	(96.7, 97.5)	96.3	97.7	0.3	(0.2, 0.3)
	6/10-yearly	7.3 (0.0)	284	(254, 318)	95.8	(95.3, 96.3)	95.1	96.5	0.8	(0.6, 1.0)
	30, 40, 50, 65	4.2 (0.0)	455	(415, 498)	93.3	(92.7, 93.9)	92.3	94.1	1.0	(0.5, 1.5)
	25, 35, 50, 65	4.3 (0.0)	475	(433, 519)	93.0	(92.4, 93.6)	91.9	94.0	0.6	(0.1, 1.2)
	30, 40, 55	3.2 (0.0)	521	(477, 566)	92.4	(91.7, 93.0)	91.2	93.4	2.6	(2.1, 3.2)
	30, 45, 60	3.2 (0.0)	577	(533, 623)	91.6	(90.8, 92.2)	90.5	92.5	1.9	(1.4, 2.5)
	25, 45, 65	3.2 (0.0)	739	(685, 794)	89.2	(88.3, 90.0)	87.6	90.7	-0.4	(-1.1, 0.2)
	30, 45	2.2 (0.0)	708	(654, 761)	89.6	(88.8, 90.4)	88.4	90.8	3.9	(3.3, 4.7)
	30, 55	2.2 (0.0)	779	(724, 837)	88.6	(87.7, 89.4)	87.1	89.9	3.0	(2.3, 3.7)
	35	1.1 (0.0)	1000	(938, 1068)	85.4	(84.3, 86.3)	83.5	87.2	14.3	(13.4, 15.3)
	40	1.1 (0.0)	1048	(983, 1114)	84.7	(83.7, 85.7)	83.1	86.4	13.9	(13.0, 14.8)
	30	1.1 (0.0)	1063	(998, 1126)	84.4	(83.4, 85.4)	82.9	86.2	12.7	(11.9, 13.5)
	45	1.0 (0.0)	1225	(1159, 1298)	82.1	(80.9, 83.1)	79.9	84.1	11.7	(10.9, 12.6)
II	None		1778	(1698, 1862)	74.0	(72.6, 75.3)	71.7	76.2		
	3/5-yearly	12.3 (0.0)	169	(145, 195)	97.5	(97.1, 97.9)	97.0	98.1	0.2	(0.1, 0.3)
	6/10-yearly	7.2 (0.0)	246	(216, 276)	96.4	(96.0, 96.8)	95.7	97.2	0.7	(0.5, 0.9)
	30, 40, 50, 65	4.2 (0.0)	392	(355, 431)	94.3	(93.7, 94.8)	93.2	95.2	0.9	(0.4, 1.4)
	25, 35, 50, 65	4.3 (0.0)	411	(371, 453)	94.0	(93.3, 94.6)	93.0	95.0	0.5	(0.0, 1.1)
	30, 40, 55	3.2 (0.0)	451	(411, 490)	93.4	(92.8, 94.0)	92.4	94.4	2.3	(1.7, 2.8)
	30, 45, 60	3.1 (0.0)	499	(457, 542)	92.7	(92.0, 93.3)	91.7	93.6	1.6	(1.1, 2.2)
	25, 45, 65	3.1 (0.0)	639	(593, 688)	90.7	(89.9, 91.4)	89.5	91.8	-0.4	(-1.0, 0.2)

	30, 45	2.1 (0.0)	612	(567, 658)	91.0	(90.3, 91.7)	89.9	92.1	3.5	(2.9, 4.1)
	30, 55	2.1 (0.0)	674	(627, 723)	90.1	(89.4, 90.8)	89.1	91.4	2.6	(2.0, 3.2)
	35	1.1 (0.0)	865	(809, 919)	87.3	(86.5, 88.2)	86.0	88.7	12.6	(11.7, 13.4)
	40	1.0 (0.0)	906	(850, 964)	86.7	(85.8, 87.6)	85.3	88.1	12.2	(11.3, 13.1)
	30	1.1 (0.0)	917	(861, 977)	86.6	(85.6, 87.5)	85.1	87.8	11.2	(10.4, 12.0)
	45	1.0 (0.0)	1061	(999, 1124)	84.5	(83.5, 85.5)	83.0	86.3	10.2	(9.4, 11.0)
III	None		1628	(1548, 1710)	76.2	(74.9, 77.4)	74.0	78.3		
	3/5-yearly	12.3 (0.0)	154	(130, 177)	97.8	(97.4, 98.1)	97.2	98.3	0.2	(0.1, 0.3)
	6/10-yearly	7.2 (0.0)	224	(194, 253)	96.7	(96.3, 97.2)	96.0	97.4	0.6	(0.5, 0.8)
	30, 40, 50, 65	4.1 (0.0)	359	(325, 399)	94.7	(94.2, 95.2)	93.6	95.6	0.8	(0.3, 1.3)
	25, 35, 50, 65	4.2 (0.0)	376	(336, 417)	94.5	(93.9, 95.1)	93.4	95.4	0.5	(0.0, 1.0)
	30, 40, 55	3.2 (0.0)	413	(375, 454)	94.0	(93.4, 94.5)	93.0	95.0	2.1	(1.6, 2.6)
	30, 45, 60	3.1 (0.0)	457	(418, 499)	93.3	(92.7, 93.9)	92.4	94.3	1.5	(1.0, 2.0)
	25, 45, 65	3.1 (0.0)	585	(538, 633)	91.4	(90.7, 92.1)	90.2	92.8	-0.4	(-1.0, 0.2)
	30, 45	2.1 (0.0)	559	(513, 608)	91.8	(91.1, 92.5)	90.8	92.8	3.2	(2.6, 3.8)
	30, 55	2.1 (0.0)	616	(569, 666)	91.0	(90.2, 91.7)	89.8	92.1	2.4	(1.8, 3.0)
	35	1.1 (0.0)	790	(735, 843)	88.4	(87.6, 89.3)	86.9	89.9	11.6	(10.7, 12.4)
	40	1.0 (0.0)	830	(772, 885)	87.9	(87.0, 88.7)	86.4	89.4	11.2	(10.4, 12.1)
	30	1.1 (0.0)	840	(783, 898)	87.7	(86.8, 88.6)	86.3	89.1	10.4	(9.6, 11.2)
	45	1.0 (0.0)	972	(911, 1038)	85.8	(84.9, 86.7)	84.1	87.2	9.4	(8.6, 10.2)
IV	None		2377	(2282, 2472)	65.2	(63.6, 66.8)	62.5	67.5		
	3/5-yearly	12.4 (0.0)	241	(211, 274)	96.5	(96.0, 96.9)	95.6	97.2	0.3	(0.2, 0.4)
	6/10-yearly	7.3 (0.0)	347	(308, 385)	94.9	(94.4, 95.5)	94.1	95.7	0.9	(0.8, 1.1)
	30, 40, 50, 65	4.2 (0.0)	546	(504, 593)	92.0	(91.3, 92.6)	90.7	93.0	1.4	(0.8, 2.0)
	25, 35, 50, 65	4.3 (0.0)	578	(532, 623)	91.5	(90.8, 92.2)	90.3	92.6	0.8	(0.2, 1.4)
	30, 40, 55	3.2 (0.0)	637	(588, 688)	90.7	(89.9, 91.4)	89.3	92.0	3.2	(2.5, 3.8)
	30, 45, 60	3.2 (0.0)	692	(639, 744)	89.9	(89.1, 90.7)	88.6	91.0	2.5	(1.9, 3.1)
	25, 45, 65	3.2 (0.0)	887	(824, 944)	87.0	(86.1, 88.0)	85.5	88.6	-0.3	(-1.1, 0.4)
	30, 45	2.2 (0.0)	865	(807, 921)	87.3	(86.5, 88.3)	85.7	88.8	4.6	(3.9, 5.3)
	30, 55	2.2 (0.0)	939	(880, 1000)	86.3	(85.3, 87.2)	84.6	87.6	3.6	(2.8, 4.3)
	35	1.1 (0.0)	1207	(1139, 1275)	82.3	(81.3, 83.4)	80.6	83.9	15.9	(14.9, 16.8)
	40	1.1 (0.0)	1249	(1182, 1320)	81.7	(80.6, 82.8)	79.9	83.6	15.5	(14.6, 16.6)
	30	1.2 (0.0)	1290	(1223, 1361)	81.1	(80.0, 82.2)	79.2	82.7	13.8	(12.9, 14.8)
	45	1.0 (0.0)	1439	(1367, 1513)	78.9	(77.8, 80.1)	77.3	80.6	13.3	(12.3, 14.2)
	None		807	(749, 864)	88.2	(87.3, 89.1)	86.6	89.6		
	3/5-yearly	12.1 (0.0)	75	(59, 93)	98.9	(98.6, 99.1)	98.4	99.3	0.1	(0.0, 0.2)
	6/10-yearly	7.1 (0.0)	109	(89, 130)	98.4	(98.1, 98.7)	97.8	98.9	0.3	(0.2, 0.4)
	30, 40, 50, 65	4.1 (0.0)	176	(150, 202)	97.4	(97.0, 97.8)	96.8	98.1	0.4	(0.1, 0.7)
	25, 35, 50, 65	4.1 (0.0)	185	(158, 212)	97.3	(96.9, 97.7)	96.7	97.9	0.2	(-0.1, 0.6)

V	30, 40, 55	3.1 (0.0)	203	(176, 230)	97.0	(96.6, 97.4)	96.4	97.7	1.0	(0.7, 1.4)
	30, 45, 60	3.1 (0.0)	224	(195, 254)	96.7	(96.3, 97.2)	96.0	97.5	0.7	(0.4, 1.1)
	25, 45, 65	3.1 (0.0)	288	(254, 323)	95.8	(95.3, 96.3)	95.0	96.7	-0.2	(-0.6, 0.2)
	30, 45	2.1 (0.0)	274	(240, 307)	96.0	(95.5, 96.5)	95.2	96.8	1.6	(1.2, 2.1)
	30, 55	2.1 (0.0)	303	(269, 337)	95.6	(95.1, 96.1)	94.8	96.4	1.2	(0.7, 1.7)
	35	1.0 (0.0)	389	(350, 430)	94.3	(93.7, 94.9)	93.3	95.4	6.0	(5.4, 6.6)
	40	1.0 (0.0)	409	(368, 447)	94.0	(93.4, 94.7)	93.1	95.2	5.7	(5.1, 6.3)
	30	1.1 (0.0)	412	(370, 453)	94.0	(93.3, 94.6)	92.8	95.0	5.5	(4.9, 6.1)
	45	1.0 (0.0)	480	(438, 522)	93.0	(92.3, 93.6)	91.8	94.0	4.7	(4.2, 5.3)

Central Range, the 2.5th and 97.5th percentiles

Vaccine I: 100% efficacy against HPV16/18, no cross protection or waning efficacy

Vaccine II: 100% efficacy against HPV16/18, cross protection for the quadrivalent vaccine, preventing 14.7% of other high risk HPV infections

Vaccine III: 100% efficacy against HPV16/18, cross protection for the bivalent vaccine, preventing 22.1% of other high risk HPV infections

Vaccine IV: 100% initial efficacy against HPV16/18, but efficacy wanes by 0.25% (absolute) every 6 months

Vaccine V: the nonavalent vaccine, providing 100% efficacy against HPV16/18 and preventing 63.6% of other high risk HPV infections

*Screening scenarios provide screening ages, except 3/5 (6/10)-yearly represents screening every 3(6) years aged 25-49 years and every 5(10) years aged 50-64 years.

Table S4: Number of screens, cancers, reduction in cancer incidence and incremental benefit (the reduction in cancer incidence per additional screen) for realistic screening coverage, for each combination of vaccination and screening scenario, from 1,000 simulated datasets of 300,000 women with fixed natural history parameters given in Figure 1

Vaccine	Screening scenario*	Number of screens	Number of cancers (per cohort of 300,000)		Cancers prevented				Incremental benefit	
		Mean (sd)	Mean	95% CR	%	95% CR	Minimum	Maximum	%	95% CR
None	None		6835	(6680, 6994)						
	HPV primary testing									
	3/5-yearly	7.2 (0.0)	1955	(1867, 2039)	71.4	(70.3, 72.5)	69.7	72.8	0.9	(0.3, 1.6)
	6/10-yearly	5.1 (0.0)	2087	(2001, 2173)	69.5	(68.4, 70.5)	67.6	71.4	3.0	(2.3, 3.7)
	30, 40, 50, 65	3.2 (0.0)	2467	(2371, 2560)	63.9	(62.8, 65.1)	62.0	66.0	2.9	(0.5, 5.4)
	30, 40, 55	2.7 (0.0)	2575	(2482, 2673)	62.3	(61.1, 63.5)	60.1	64.6	23.2	(22.8, 23.7)
	Cytology primary testing									
	3/5-yearly	6.6 (0.0)	2632	(2530, 2725)	61.5	(60.3, 62.6)	59.3	63.6	1.6	(0.9, 2.3)
	6/10-yearly	4.6 (0.0)	2850	(2754, 2951)	58.3	(57.2, 59.4)	56.2	60.5	12.8	(12.5, 13.0)
I	None		2050	(1961, 2142)	70.0	(68.5, 71.4)	67.5	72.4		
	3/5-yearly	6.9 (0.0)	609	(561, 657)	91.1	(90.4, 91.8)	89.8	92.3	0.3	(-0.1, 0.6)
	6/10-yearly	4.8 (0.0)	648	(598, 699)	90.5	(89.7, 91.2)	89.1	91.6	0.9	(0.5, 1.4)
	30, 40, 50, 65	3.1 (0.0)	756	(703, 812)	88.9	(88.1, 89.7)	87.7	90.1	1.0	(-0.3, 2.4)
	25, 35, 50, 65	3.2 (0.0)	774	(719, 832)	88.7	(87.8, 89.5)	87.2	90.0	0.5	(-0.7, 1.6)
	30, 40, 55	2.5 (0.0)	795	(738, 854)	88.4	(87.4, 89.2)	86.7	89.7	2.7	(1.9, 3.6)
	30, 45, 60	2.4 (0.0)	844	(787, 901)	87.6	(86.7, 88.5)	86.3	88.8	2.2	(1.2, 3.3)
	25, 45, 65	2.3 (0.0)	976	(911, 1044)	85.7	(84.6, 86.7)	84.1	87.4	-0.5	(-1.8, 0.9)
	30, 45	1.7 (0.0)	956	(898, 1019)	86.0	(85.0, 86.9)	84.5	87.7	3.9	(2.9, 5.0)
	30, 55	1.8 (0.0)	985	(924, 1050)	85.6	(84.5, 86.5)	84.1	87.1	2.9	(1.9, 3.8)
	35	0.9 (0.0)	1159	(1089, 1228)	83.0	(81.9, 84.1)	81.0	84.7	14.1	(13.1, 15.2)
	40	0.9 (0.0)	1217	(1147, 1289)	82.2	(81.0, 83.3)	79.8	83.8	13.4	(12.5, 14.4)
	30	1.0 (0.0)	1196	(1129, 1263)	82.5	(81.4, 83.5)	80.5	84.1	12.8	(11.9, 13.9)
	45	0.9 (0.0)	1367	(1291, 1440)	80.0	(78.9, 81.1)	77.3	81.8	11.2	(10.3, 12.1)
	None		1778	(1698, 1862)	74.0	(72.6, 75.3)	71.7	76.2		
	3/5-yearly	6.8 (0.0)	527	(487, 574)	92.3	(91.6, 92.9)	91.3	93.2	0.2	(-0.1, 0.6)
	6/10-yearly	4.8 (0.0)	561	(517, 609)	91.8	(91.1, 92.4)	90.7	92.8	0.8	(0.4, 1.2)
	30, 40, 50, 65	3.1 (0.0)	655	(603, 704)	90.4	(89.7, 91.2)	89.1	91.8	0.9	(-0.4, 2.2)

II	25, 35, 50, 65	3.2 (0.0)	689	(624, 721)	90.2	(89.4, 90.9)	88.8	91.4	0.4	(-0.8, 1.6)
	30, 40, 55	2.5 (0.0)	670	(638, 741)	89.9	(89.2, 90.7)	88.7	91.1	2.4	(1.6, 3.2)
	30, 45, 60	2.4 (0.0)	731	(679, 782)	89.3	(88.5, 90.1)	88.2	90.5	1.9	(1.0, 2.9)
	25, 45, 65	2.3 (0.0)	845	(790, 902)	87.6	(86.8, 88.5)	86.0	88.9	-0.4	(-1.6, 0.8)
	30, 45	1.7 (0.0)	827	(771, 882)	87.9	(87.1, 88.8)	86.6	89.3	3.5	(2.5, 4.5)
	30, 55	1.8 (0.0)	853	(797, 912)	87.5	(86.6, 88.3)	86.0	88.8	2.5	(1.7, 3.4)
	35	0.9 (0.0)	1004	(945, 1063)	85.3	(84.4, 86.3)	83.7	86.7	12.4	(11.4, 13.3)
	40	0.9 (0.0)	1054	(990, 1120)	84.6	(83.5, 85.6)	82.7	86.5	11.7	(10.8, 12.7)
	30	1.0 (0.0)	1034	(972, 1095)	84.9	(83.9, 85.8)	83.3	86.6	11.4	(10.5, 12.3)
	45	0.9 (0.0)	1185	(1115, 1253)	82.7	(81.6, 83.7)	81.0	84.2	9.8	(8.9, 10.7)
III	None		1628	(1548, 1710)	76.2	(74.9, 77.4)	74.0	78.3		
	3/5-yearly	6.8 (0.0)	483	(440, 522)	92.9	(92.3, 93.6)	92.0	93.8	0.2	(-0.1, 0.5)
	6/10-yearly	4.7 (0.0)	513	(221, 284)	92.5	(91.9, 93.1)	91.3	93.6	0.7	(0.4, 1.1)
	30, 40, 50, 65	3.0 (0.0)	601	(554, 650)	91.2	(90.5, 91.9)	90.1	92.5	0.8	(-0.4, 2.0)
	25, 35, 50, 65	3.1 (0.0)	612	(566, 660)	91.0	(90.3, 91.7)	89.9	92.1	0.4	(-0.7, 1.5)
	30, 40, 55	2.5 (0.0)	629	(582, 680)	90.8	(90.0, 91.5)	89.5	91.9	2.2	(1.3, 3.0)
	30, 45, 60	2.4 (0.0)	669	(620, 718)	90.2	(89.5, 91.0)	89.0	91.4	1.7	(0.9, 2.6)
	25, 45, 65	2.2 (0.0)	773	(722, 826)	88.7	(87.9, 89.5)	87.3	90.0	-0.4	(-1.6, 0.8)
	30, 45	1.6 (0.0)	756	(703, 810)	88.9	(88.1, 89.7)	87.7	90.2	3.2	(2.2, 4.0)
	30, 55	1.7 (0.0)	780	(727, 839)	88.6	(87.7, 89.4)	87.3	89.8	2.3	(1.5, 3.1)
	35	0.9 (0.0)	917	(859, 975)	86.6	(85.7, 87.5)	85.0	88.0	11.4	(10.5, 12.3)
	40	0.9 (0.0)	964	(903, 1028)	85.9	(84.9, 86.8)	84.2	87.3	10.8	(9.9, 11.8)
	30	0.9 (0.0)	946	(887, 1010)	86.2	(85.2, 87.1)	84.6	87.6	10.5	(9.6, 11.4)
	45	0.9 (0.0)	1084	(1022, 1149)	84.1	(83.1, 85.1)	82.5	85.6	9.0	(8.2, 9.8)
IV	None		2377	(2282, 2472)	65.2	(63.6, 66.8)	62.5	67.5		
	3/5-yearly	6.9 (0.0)	733	(685, 787)	89.3	(88.4, 90.0)	87.6	90.6	0.3	(-0.1, 0.7)
	6/10-yearly	4.9 (0.0)	775	(722, 826)	88.7	(87.9, 89.5)	87.2	89.9	1.0	(0.6, 1.5)
	30, 40, 50, 65	3.1 (0.0)	900	(844, 959)	86.8	(85.9, 87.7)	85.2	88.3	1.3	(-0.2, 2.7)
	25, 35, 50, 65	3.2 (0.0)	926	(868, 980)	86.4	(85.6, 87.3)	85.1	87.9	0.5	(-0.6, 1.8)
	30, 40, 55	2.6 (0.0)	950	(892, 1011)	86.1	(85.2, 87.0)	84.4	87.4	3.3	(2.4, 4.3)
	30, 45, 60	2.4 (0.0)	1003	(945, 1069)	85.3	(84.3, 86.2)	83.5	86.8	2.8	(1.8, 4.0)
	25, 45, 65	2.3 (0.0)	1163	(1098, 1230)	83.0	(81.9, 84.0)	80.8	84.9	-0.4	(-1.7, 1.1)
	30, 45	1.7 (0.0)	1147	(1083, 1214)	83.2	(82.2, 84.3)	81.1	84.8	4.5	(3.4, 5.7)

	30, 55	1.8 (0.0)	1172	(1105, 1239)	82.9	(81.8, 84.0)	81.2	84.6	3.5	(2.5, 4.5)
	35	0.9 (0.0)	1384	(1313, 1462)	79.7	(78.5, 81.0)	78.0	81.6	15.6	(14.6, 16.7)
	40	0.9 (0.0)	1439	(1367, 1516)	78.9	(77.7, 80.1)	77.2	80.7	15.0	(13.9, 16.1)
	30	1.0 (0.0)	1436	(1363, 1505)	79.0	(77.8, 80.1)	76.8	80.8	14.0	(13.0, 15.0)
	45	0.9 (0.0)	1601	(1526, 1677)	76.6	(75.3, 77.8)	74.6	78.4	12.7	(11.7, 13.7)
V	None		807	(749, 864)	88.2	(87.3, 89.1)	86.6	89.6		
	3/5-yearly	6.7 (0.0)	238	(207, 269)	96.5	(96.0, 97.0)	95.6	97.2	0.1	(-0.1, 0.3)
	6/10-yearly	4.7 (0.0)	253	(221, 284)	96.3	(95.8, 96.8)	95.5	97.1	0.4	(0.1, 0.7)
	30, 40, 50, 65	3.0 (0.0)	296	(259, 329)	95.7	(95.2, 96.2)	94.8	96.6	0.4	(-0.5, 1.2)
	25, 35, 50, 65	3.1 (0.0)	302	(267, 337)	95.6	(95.1, 96.1)	94.8	96.4	0.2	(-0.6, 1.1)
	30, 40, 55	2.5 (0.0)	310	(273, 346)	95.5	(94.9, 96.0)	94.6	96.3	1.1	(0.5, 1.7)
	30, 45, 60	2.4 (0.0)	329	(295, 366)	95.2	(94.6, 95.7)	94.2	96.2	0.9	(0.2, 1.5)
	25, 45, 65	2.2 (0.0)	381	(344, 419)	94.4	(93.8, 95.0)	93.5	95.3	-0.2	(-1.0, 0.6)
	30, 45	1.6 (0.0)	373	(335, 410)	94.5	(94.0, 95.1)	93.6	95.6	1.6	(1.0, 2.3)
	30, 55	1.7 (0.0)	384	(348, 423)	94.4	(93.8, 94.9)	93.5	95.3	1.2	(0.5, 1.8)
	35	0.9 (0.0)	452	(409, 494)	93.4	(92.8, 94.0)	92.3	94.3	5.9	(5.2, 6.5)
	40	0.9 (0.0)	476	(433, 518)	93.0	(92.4, 93.7)	92.0	94.1	5.5	(4.9, 6.2)
	30	0.9 (0.0)	466	(421, 512)	93.2	(92.5, 93.9)	92.1	94.1	5.5	(4.9, 6.1)
	45	0.9 (0.0)	537	(490, 584)	92.1	(91.4, 92.8)	90.9	93.2	4.5	(4.0, 5.1)

Central Range, the 2.5th and 97.5th percentiles

Vaccine I: 100% efficacy against HPV16/18, no cross protection or waning efficacy

Vaccine II: 100% efficacy against HPV16/18, cross protection for the quadrivalent vaccine, preventing 14.7% of other high risk HPV infections

Vaccine III: 100% efficacy against HPV16/18, cross protection for the bivalent vaccine, preventing 22.1% of other high risk HPV infections

Vaccine IV: 100% initial efficacy against HPV16/18, but efficacy wanes by 0.25% (absolute) every 6 months

Vaccine V: the nonavalent vaccine, providing 100% efficacy against HPV16/18 and preventing 63.6% of other high risk HPV infections

*Screening scenarios provide screening ages, except 3/5 (6/10)-yearly represents screening every 3(6) years aged 25-49 years and every 5(10) years aged 50-64 years.

Table S5: Number of screens, cancers, reduction in cancer incidence and incremental benefit (the reduction in cancer incidence per additional screen) for 100% screening coverage, for each combination of vaccination and screening scenario, from simulated datasets of 300,000 women with natural history parameters drawn from the distributions given in Figure 1 which had a lifetime risk of cervical cancer in the absence of vaccination or screening ≤1.7%

Vaccine	Screening scenario*	Number of screens	Number of cancers (per cohort of 300,000)		Cancers prevented				Incremental benefit	
		Mean (sd)	Mean	95% CR	%	95% CR	Minimum	Maximum	%	95% CR
None	None		4759	(4240, 4995)						
	HPV primary testing									
	3/5-yearly	12.7 (0.0)	499	(405, 568)	89.5	(88.2, 91.1)	86.9	92.1	0.9	(0.7, 1.2)
	6/10-yearly	7.5 (0.1)	729	(610, 826)	84.7	(82.6, 86.8)	81.0	87.8	2.8	(2.3, 3.2)
	30, 40, 50, 65	4.4 (0.0)	1152	(984, 1279)	75.8	(73.1, 78.5)	71.0	80.3	3.3	(2.1, 4.6)
	30, 40, 55	3.4 (0.0)	1307	(1122, 1445)	72.5	(70.0, 76.0)	66.9	77.7	21.4	(20.5, 22.3)
	Cytology primary testing									
	3/5-yearly	12.0 (0.0)	732	(603, 835)	84.5	(82.4, 86.7)	81.2	88.5	1.7	(1.4, 2.0)
	6/10-yearly	7.0 (0.0)	1130	(962, 1267)	76.0	(73.4, 79.3)	70.5	81.0	10.9	(10.5, 11.3)
I	None		1440	(1144, 1721)	69.7	(64.8, 75.0)	63.7	76.8		
	3/5-yearly	12.3 (0.0)	166	(124, 211)	96.5	(95.5, 97.4)	95.2	97.8	0.3	(0.2, 0.4)
	6/10-yearly	7.3 (0.0)	234	(178, 286)	95.1	(93.7, 96.2)	93.4	96.7	0.9	(0.6, 1.1)
	30, 40, 50, 65	4.2 (0.0)	360	(274, 436)	92.4	(90.8, 94.1)	90.4	94.8	1.0	(0.5, 1.6)
	25, 35, 50, 65	4.3 (0.0)	380	(291, 459)	92.0	(90.4, 93.8)	90.0	94.7	0.5	(-0.2, 1.2)
	30, 40, 55	3.2 (0.0)	406	(319, 481)	91.5	(89.6, 93.2)	89.0	94.1	2.6	(1.8, 3.7)
	30, 45, 60	3.2 (0.0)	448	(356, 541)	90.6	(88.9, 92.5)	88.3	93.2	1.8	(1.0, 2.6)
	25, 45, 65	3.2 (0.0)	557	(445, 670)	88.3	(86.0, 90.4)	85.6	91.6	-0.5	(-1.4, 0.4)
	30, 45	2.1 (0.0)	535	(427, 658)	88.8	(86.6, 90.9)	86.0	91.5	4.0	(2.8, 5.0)
	30, 55	2.2 (0.0)	592	(473, 712)	87.5	(85.3, 89.9)	84.6	91.0	2.9	(1.8, 4.0)
	35	1.1 (0.0)	743	(599, 899)	84.4	(81.6, 87.3)	80.3	88.6	13.7	(11.1, 16.6)
	40	1.1 (0.0)	788	(621, 944)	83.4	(80.4, 86.4)	78.8	87.5	13.0	(10.1, 15.6)
	30	1.1 (0.0)	778	(620, 939)	83.6	(80.8, 87.0)	79.2	87.8	12.3	(9.8, 14.7)
	45	1.0 (0.0)	905	(729, 1075)	81.0	(77.8, 84.3)	76.9	85.2	10.9	(8.3, 13.1)
	None		1256	(992, 1499)	73.6	(69.2, 78.5)	68.4	79.1		
	3/5-yearly	12.3 (0.0)	130	(96, 165)	97.3	(96.6, 97.9)	95.7	98.1	0.3	(0.2, 0.4)
	6/10-yearly	7.2 (0.0)	190	(142, 246)	96.0	(94.9, 96.9)	94.3	97.2	0.8	(0.6, 1.1)

II	30, 40, 50, 65	4.2 (0.0)	311	(241, 377)	93.5	(92.2, 95.0)	91.0	95.8	0.8	(0.2, 1.4)
	25, 35, 50, 65	4.3 (0.0)	320	(248, 392)	93.3	(91.7, 94.8)	91.3	95.6	0.6	(-0.1, 1.1)
	30, 40, 55	3.2 (0.0)	350	(319, 481)	92.6	(91.0, 94.2)	90.6	95.2	2.2	(1.5, 2.9)
	30, 45, 60	3.1 (0.0)	379	(299, 465)	92.0	(90.4, 93.8)	89.4	94.6	1.6	(0.9, 2.3)
	25, 45, 65	3.1 (0.0)	473	(378, 573)	90.0	(88.1, 92.0)	87.3	92.6	-0.3	(-1.2, 0.4)
	30, 45	2.1 (0.0)	457	(368, 550)	90.4	(88.4, 92.5)	87.5	93.6	3.7	(2.8, 4.7)
	30, 55	2.1 (0.0)	505	(404, 615)	89.4	(87.3, 91.4)	86.1	92.7	2.7	(1.7, 3.8)
	35	1.1 (0.0)	644	(515, 779)	86.5	(83.9, 89.2)	83.0	90.2	12.1	(9.6, 14.9)
	40	1.0 (0.0)	683	(553, 826)	85.6	(83.0, 88.2)	82.2	89.5	11.5	(9.1, 13.7)
	30	1.1 (0.0)	675	(534, 806)	85.8	(83.2, 88.5)	81.9	89.8	10.9	(8.6, 13.5)
	45	1.0 (0.0)	777	(615, 938)	83.7	(80.7, 86.9)	80.4	87.5	9.8	(7.7, 11.6)
III	None		1143	(913, 1359)	76.0	(72.1, 80.4)	70.9	81.5		
	3/5-yearly	12.2 (0.0)	121	(93, 148)	97.5	(96.9, 98.0)	96.6	98.4	0.2	(0.1, 0.3)
	6/10-yearly	7.2 (0.0)	174	(134, 211)	96.3	(95.6, 97.1)	95.4	97.5	0.7	(0.5, 1.0)
	30, 40, 50, 65	4.1 (0.0)	278	(219, 334)	94.1	(93.0, 95.4)	92.7	95.8	0.8	(0.2, 1.3)
	25, 35, 50, 65	4.2 (0.0)	286	(227, 348)	94.0	(92.8, 95.2)	92.4	95.6	0.6	(-0.1, 1.3)
	30, 40, 55	3.2 (0.0)	316	(246, 374)	93.4	(92.2, 94.8)	91.5	95.1	2.3	(2.4, 4.2)
	30, 45, 60	3.1 (0.0)	348	(283, 416)	92.7	(91.3, 94.1)	91.0	94.5	1.6	(0.9, 2.5)
	25, 45, 65	3.1 (0.0)	444	(359, 540)	90.7	(89.0, 92.4)	88.4	93.0	-0.4	(-1.2, 0.3)
	30, 45	2.1 (0.0)	427	(345, 517)	91.0	(89.3, 92.7)	88.9	93.3	3.0	(2.0, 4.0)
	30, 55	2.1 (0.0)	462	(371, 554)	90.3	(88.5, 92.1)	87.8	92.4	2.3	(1.2, 3.3)
	35	1.1 (0.0)	576	(453, 699)	87.9	(85.7, 90.2)	84.9	90.9	11.3	(8.8, 13.5)
	40	1.0 (0.0)	611	(495, 719)	87.2	(85.2, 89.5)	84.0	90.0	10.7	(8.6, 13.1)
	30	1.1 (0.0)	612	(482, 742)	87.1	(84.9, 89.7)	83.6	90.2	10.1	(8.1, 12.0)
	45	1.0 (0.0)	719	(574, 860)	84.9	(82.4, 87.7)	81.3	88.5	8.7	(6.7, 10.7)
IV	None		1681	(1370, 1937)	64.7	(60.2, 70.2)	58.8	72.3		
	3/5-yearly	12.3 (0.0)	199	(155, 237)	95.8	(95.0, 96.7)	94.6	97.3	0.3	(0.2, 0.4)
	6/10-yearly	7.3 (0.0)	277	(220, 326)	94.2	(93.1, 95.4)	92.6	96.1	1.1	(0.8, 1.3)
	30, 40, 50, 65	4.2 (0.0)	435	(339, 515)	90.8	(89.4, 92.6)	88.8	93.6	1.4	(0.5, 2.2)
	25, 35, 50, 65	4.3 (0.0)	500	(356, 528)	90.6	(89.0, 92.5)	88.3	93.1	1.0	(0.3, 1.7)
	30, 40, 55	3.2 (0.0)	448	(393, 586)	89.5	(87.8, 91.5)	87.1	92.4	3.3	(1.5, 3.1)
	30, 45, 60	3.2 (0.0)	533	(427, 624)	88.8	(87.0, 90.9)	86.5	91.8	2.7	(1.9, 3.6)
	25, 45, 65	3.2 (0.0)	672	(555, 787)	85.9	(83.5, 88.3)	82.8	89.2	-0.2	(-1.1, 0.8)

	30, 45	2.2 (0.0)	664	(547, 778)	86.0	(83.9, 88.4)	82.6	89.9	4.3	(3.1, 5.6)
	30, 55	2.2 (0.0)	707	(563, 817)	85.1	(83.0, 87.7)	82.3	88.9	3.5	(2.2, 4.7)
	35	1.1 (0.0)	887	(719, 1022)	81.3	(78.7, 84.6)	77.6	85.6	15.5	(12.9, 18.2)
	40	1.1 (0.0)	938	(749, 1092)	80.3	(77.4, 83.7)	75.9	85.2	14.7	(12.2, 17.3)
	30	1.1 (0.0)	936	(779, 1071)	80.3	(77.2, 83.4)	75.9	85.0	13.7	(11.3, 15.9)
	45	1.0 (0.0)	1080	(864, 1249)	77.3	(74.1, 81.1)	73.2	82.9	12.2	(9.8, 14.6)
V	None		569	(447, 674)	88.0	(86.1, 90.4)	85.5	91.9		
	3/5-yearly	12.1 (0.0)	66	(46, 86)	98.6	(98.2, 99.0)	98.0	99.2	0.1	(0.1, 0.2)
	6/10-yearly	7.1 (0.0)	96	(70, 122)	98.0	(97.4, 98.5)	97.3	98.7	0.4	(0.2, 0.5)
	30, 40, 50, 65	4.1 (0.0)	148	(114, 182)	96.9	(96.2, 97.6)	95.9	98.0	0.4	(-0.0, 0.8)
	25, 35, 50, 65	4.1 (0.0)	149	(115, 186)	96.9	(96.2, 97.5)	96.1	97.8	0.4	(-0.1, 0.8)
	30, 40, 55	3.1 (0.0)	167	(127, 203)	96.5	(95.8, 97.3)	95.5	97.7	1.0	(0.6, 1.4)
	30, 45, 60	3.1 (0.0)	179	(132, 218)	96.2	(95.4, 97.1)	95.0	97.5	0.7	(0.3, 1.2)
	25, 45, 65	3.1 (0.0)	219	(170, 265)	95.4	(94.5, 96.4)	94.0	97.2	-0.1	(-0.6, 0.4)
	30, 45	2.1 (0.0)	215	(168, 263)	95.5	(94.6, 96.4)	94.3	97.1	1.6	(1.0, 2.2)
	30, 55	2.1 (0.0)	236	(179, 286)	95.0	(94.1, 96.1)	93.7	96.6	1.1	(0.5, 1.9)
	35	1.0 (0.0)	291	(224, 353)	93.9	(92.7, 95.1)	92.3	95.6	5.7	(4.4, 6.8)
	40	1.0 (0.0)	313	(241, 378)	93.4	(92.2, 94.7)	91.9	95.3	5.3	(4.0, 6.3)
	30	1.1 (0.0)	302	(234, 372)	93.6	(92.4, 94.9)	92.0	95.4	5.3	(4.1, 6.5)
	45	1.0 (0.0)	365	(279, 438)	92.3	(90.9, 93.9)	90.6	94.6	4.2	(3.1, 5.2)

Central Range, the 2.5th and 97.5th percentiles

Vaccine I: 100% efficacy against HPV16/18, no cross protection or waning efficacy

Vaccine II: 100% efficacy against HPV16/18, cross protection for the quadrivalent vaccine, preventing 14.7% of other high risk HPV infections

Vaccine III: 100% efficacy against HPV16/18, cross protection for the bivalent vaccine, preventing 22.1% of other high risk HPV infections

Vaccine IV: 100% initial efficacy against HPV16/18, but efficacy wanes by 0.25% (absolute) every 6 months

Vaccine V: the nonavalent vaccine, providing 100% efficacy against HPV16/18 and preventing 63.6% of other high risk HPV infections

*Screening scenarios provide screening ages, except 3/5 (6/10)-yearly represents screening every 3(6) years aged 25-49 years and every 5(10) years aged 50-64 years.

Table S6: Number of screens, cancers, reduction in cancer incidence and incremental benefit (the reduction in cancer incidence per additional screen) for 100% screening coverage, for each combination of vaccination and screening scenario, from simulated datasets of 300,000 women with natural history parameters drawn from the distributions given in Figure 1 which had a lifetime risk of cervical cancer in the absence of vaccination or screening ≥2.7%

Vaccine	Screening scenario*	Number of screens	Number of cancers (per cohort of 300,000)		Cancers prevented				Incremental benefit	
		Mean (sd)	Mean	95% CR	%	95% CR	Minimum	Maximum	%	95% CR
None	None		8251	(8067, 8646)						
	HPV primary testing									
	3/5-yearly	12.6 (0.0)	656	(583, 740)	92.0	(91.0, 92.9)	90.6	93.7	0.7	(0.6, 0.9)
	6/10-yearly	7.5 (0.0)	964	(854, 1063)	88.3	(87.2, 89.5)	86.3	90.6	2.5	(2.1, 2.8)
	30, 40, 50, 65	4.4 (0.0)	1607	(1451, 1735)	80.5	(79.0, 82.4)	78.0	83.5	2.7	(1.9, 3.5)
	30, 40, 55	3.4 (0.0)	1828	(1677, 1970)	77.8	(76.1, 79.6)	74.6	81.2	23.0	(22.3, 23.7)
	Cytology primary testing									
	3/5-yearly	12.0 (0.0)	954	(857, 1057)	88.3	(87.0, 89.3)	86.7	90.1	1.4	(1.3, 1.6)
	6/10-yearly	7.0 (0.0)	1538	(1404, 1663)	81.1	(79.6, 82.7)	79.3	84.2	11.6	(11.4, 11.8)
I	None		2386	(1985, 2851)	71.1	(65.8, 75.6)	64.7	76.5		
	3/5-yearly	12.3 (0.0)	210	(162, 263)	97.5	(96.8, 98.0)	96.3	98.2	0.2	(0.1, 0.3)
	6/10-yearly	7.3 (0.0)	297	(237, 371)	96.4	(95.5, 97.1)	94.9	97.3	0.7	(0.5, 0.9)
	30, 40, 50, 65	4.2 (0.0)	479	(376, 590)	94.2	(92.9, 95.4)	92.4	95.7	1.0	(0.5, 1.6)
	25, 35, 50, 65	4.3 (0.0)	507	(410, 625)	93.9	(92.3, 95.0)	91.6	95.3	0.6	(0.1, 1.2)
	30, 40, 55	3.2 (0.0)	563	(460, 691)	93.2	(91.5, 94.4)	90.9	94.8	2.5	(1.8, 3.3)
	30, 45, 60	3.2 (0.0)	623	(503, 759)	92.4	(90.8, 93.8)	90.0	94.3	1.8	(1.2, 2.5)
	25, 45, 65	3.2 (0.0)	795	(650, 961)	90.4	(88.3, 92.0)	87.6	92.6	-0.2	(-1.0, 0.5)
	30, 45	2.1 (0.0)	775	(631, 955)	90.6	(88.4, 92.3)	87.4	92.6	3.8	(2.8, 4.8)
	30, 55	2.2 (0.0)	860	(711, 1039)	89.6	(87.3, 91.3)	86.3	91.8	2.9	(2.0, 3.9)
	35	1.1 (0.0)	1116	(924, 1351)	86.5	(83.6, 88.7)	83.0	89.3	14.4	(12.0, 17.2)
	40	1.1 (0.0)	1164	(977, 1408)	85.9	(82.9, 88.2)	82.4	88.6	14.0	(11.5, 16.9)
	30	1.1 (0.0)	1201	(1000, 1448)	85.4	(82.4, 87.8)	81.2	88.5	12.7	(10.4, 15.2)
	45	1.0 (0.0)	1351	(1112, 1623)	83.6	(80.2, 86.4)	79.9	87.1	12.2	(10.0, 14.8)
	None		2098	(1725, 2517)	74.6	(69.9, 78.8)	69.0	79.8		
	3/5-yearly	12.3 (0.0)	176	(137, 223)	97.9	(97.3, 98.4)	97.1	98.4	0.2	(0.1, 0.3)
	6/10-yearly	7.2 (0.0)	256	(203, 313)	96.9	(96.3, 97.6)	96.0	97.9	0.6	(0.4, 0.8)

	30, 40, 50, 65	4.1 (0.0)	411	(330, 501)	95.0	(94.0, 96.0)	93.6	96.5	0.8	(0.4, 1.2)
	25, 35, 50, 65	4.3 (0.0)	434	(348, 540)	94.7	(93.6, 95.8)	93.1	96.1	0.4	(-0.1, 0.9)
	30, 40, 55	3.1 (0.0)	472	(377, 574)	94.3	(93.0, 95.5)	92.3	95.8	2.0	(1.5, 2.7)
II	30, 45, 60	3.1 (0.0)	527	(429, 644)	93.6	(92.2, 94.9)	91.4	95.1	1.4	(0.9, 2.1)
	25, 45, 65	3.1 (0.0)	695	(567, 847)	91.6	(89.8, 93.1)	89.1	93.4	-0.6	(-1.2, 0.1)
	30, 45	2.1 (0.0)	647	(528, 797)	92.2	(90.5, 93.7)	89.7	94.1	3.5	(2.6, 4.4)
	30, 55	2.1 (0.0)	727	(594, 905)	91.2	(89.3, 92.8)	88.2	93.4	2.6	(1.8, 3.5)
	35	1.1 (0.0)	957	(787, 1167)	88.4	(85.9, 90.5)	85.6	91.0	13.0	(10.6, 15.5)
	40	1.0 (0.0)	1000	(826, 1217)	87.9	(85.5, 90.1)	85.1	90.5	12.7	(10.4, 15.1)
	30	1.1 (0.0)	1023	(844, 1262)	87.6	(85.0, 89.8)	84.0	90.5	11.7	(9.6, 13.9)
	45	1.0 (0.0)	1182	(973, 1417)	85.7	(83.0, 88.1)	82.4	88.7	10.8	(8.9, 13.0)
	None		1914	(1590, 2272)	76.8	(72.5, 80.8)	71.7	81.7		
	3/5-yearly	12.2 (0.0)	174	(130, 218)	97.9	(97.4, 98.4)	97.2	98.5	0.2	(0.1, 0.2)
	6/10-yearly	7.2 (0.0)	245	(191, 302)	97.0	(96.3, 97.7)	96.1	97.8	0.6	(0.4, 0.7)
	30, 40, 50, 65	4.1 (0.0)	390	(319, 466)	95.3	(94.2, 96.1)	94.0	96.4	0.7	(0.3, 1.2)
	25, 35, 50, 65	4.2 (0.0)	417	(329, 508)	94.9	(93.8, 96.0)	93.4	96.2	0.4	(-0.1, 1.0)
	30, 40, 55	3.2 (0.0)	451	(371, 548)	94.5	(93.3, 95.5)	93.1	95.7	2.1	(1.4, 2.8)
	30, 45, 60	3.1 (0.0)	504	(411, 602)	93.9	(92.7, 95.0)	92.4	95.4	1.5	(0.9, 2.1)
III	25, 45, 65	3.1 (0.0)	661	(534, 796)	92.0	(90.4, 93.4)	90.0	94.0	-0.4	(-1.0, 0.2)
	30, 45	2.1 (0.0)	631	(515, 765)	92.4	(90.7, 93.8)	90.1	94.1	3.1	(2.4, 3.9)
	30, 55	2.1 (0.0)	697	(577, 842)	91.6	(89.8, 93.0)	89.4	93.5	2.3	(1.6, 3.1)
	35	1.1 (0.0)	900	(758, 1078)	89.1	(86.9, 90.8)	86.2	91.3	11.6	(9.4, 14.1)
	40	1.0 (0.0)	926	(764, 1112)	88.8	(86.7, 90.7)	85.8	91.5	11.5	(9.3, 14.0)
	30	1.1 (0.0)	976	(804, 1173)	88.2	(85.9, 90.2)	85.2	91.0	10.2	(8.4, 12.3)
	45	1.0 (0.0)	1087	(904, 1302)	86.8	(84.5, 89.1)	83.7	89.5	9.8	(7.9, 11.8)
	None		2746	(2326, 3206)	66.7	(61.6, 71.5)	60.2	72.3		
	3/5-yearly	12.3 (0.0)	244	(197, 297)	97.0	(96.4, 97.6)	96.0	97.9	0.2	(0.2, 0.3)
	6/10-yearly	7.3 (0.0)	344	(278, 420)	95.8	(94.9, 96.6)	94.4	97.0	0.8	(0.6, 1.0)
	30, 40, 50, 65	4.2 (0.0)	547	(458, 661)	93.4	(92.0, 94.4)	91.5	95.0	1.3	(0.8, 1.8)
	25, 35, 50, 65	4.3 (0.0)	590	(493, 694)	92.8	(91.6, 94.0)	90.9	94.2	0.7	(0.2, 1.2)
	30, 40, 55	3.2 (0.0)	650	(540, 770)	92.1	(90.8, 93.4)	89.6	93.7	3.1	(2.4, 3.9)
	30, 45, 60	3.2 (0.0)	717	(601, 850)	91.3	(89.9, 92.7)	89.3	93.2	2.4	(1.7, 3.1)
IV	25, 45, 65	3.2 (0.0)	931	(789, 1105)	88.7	(86.9, 90.4)	85.7	90.8	-0.2	(-0.9, 0.5)

	30, 45	2.2 (0.0)	915	(763, 1070)	88.9	(87.1, 90.7)	85.6	91.1	4.4	(3.4, 5.4)
	30, 55	2.2 (0.0)	987	(834, 1160)	88.0	(86.0, 89.8)	84.6	90.4	3.6	(2.6, 4.5)
	35	1.1 (0.0)	1311	(1117, 1552)	84.1	(81.4, 86.3)	80.6	86.9	16.2	(13.6, 19.0)
	40	1.1 (0.0)	1359	(1160, 1585)	83.5	(81.0, 85.9)	80.1	86.4	15.9	(13.2, 18.7)
	30	1.1 (0.0)	1410	(1202, 1661)	82.9	(80.1, 85.4)	78.7	86.1	14.1	(11.7, 16.4)
	45	1.0 (0.0)	1570	(1335, 1823)	81.0	(78.1, 83.8)	77.0	84.1	13.8	(11.5, 16.4)
V	None		954	(779, 1156)	88.4	(86.0. 90.5)	85.2	90.6		
	3/5-yearly	12.1 (0.0)	85	(60, 111)	99.0	(98.7, 99.3)	98.5	99.3	0.1	(0.0, 0.1)
	6/10-yearly	7.1 (0.0)	120	(89, 157)	98.5	(98.1, 98.9)	98.0	99.0	0.3	(0.2, 0.4)
	30, 40, 50, 65	4.1 (0.0)	196	(152, 247)	97.6	(97.0, 98.2)	96.9	98.4	0.3	(0.0, 0.6)
	25, 35, 50, 65	4.1 (0.0)	205	(158, 262)	97.5	(96.9, 98.1)	96.6	98.3	0.2	(-0.1, 0.5)
	30, 40, 55	3.1 (0.0)	223	(172, 282)	97.3	(96.6, 97.9)	96.4	98.0	1.0	(0.7, 1.5)
	30, 45, 60	3.1 (0.0)	252	(193, 317)	96.9	(96.2, 97.7)	95.8	97.9	0.7	(0.4, 1.0)
	25, 45, 65	3.1 (0.0)	322	(252, 405)	96.1	(95.1, 96.9)	94.6	97.1	-0.1	(-0.5, 0.2)
	30, 45	2.1 (0.0)	310	(247, 383)	96.2	(95.4, 97.0)	95.2	97.2	1.5	(1.1, 2.1)
	30, 55	2.1 (0.0)	345	(268, 429)	95.8	(94.8, 96.7)	94.5	96.9	1.1	(0.7, 1.7)
	35	1.0 (0.0)	441	(351, 549)	94.7	(93.4, 95.7)	93.0	96.1	6.1	(4.9, 7.4)
	40	1.0 (0.0)	460	(371, 567)	94.4	(93.1, 95.5)	92.9	95.7	5.9	(4.7, 7.2)
	30	1.1 (0.0)	478	(381, 595)	94.2	(92.9, 95.3)	92.6	95.6	5.5	(4.4, 6.7)
	45	1.0 (0.0)	544	(443, 663)	93.4	(92.1, 94.6)	91.6	94.9	4.9	(3.9, 6.1)

Central Range, the 2.5th and 97.5th percentiles

Vaccine I: 100% efficacy against HPV16/18, no cross protection or waning efficacy

Vaccine II: 100% efficacy against HPV16/18, cross protection for the quadrivalent vaccine, preventing 14.7% of other high risk HPV infections

Vaccine III: 100% efficacy against HPV16/18, cross protection for the bivalent vaccine, preventing 22.1% of other high risk HPV infections

Vaccine IV: 100% initial efficacy against HPV16/18, but efficacy wanes by 0.25% (absolute) every 6 months

Vaccine V: the nonavalent vaccine, providing 100% efficacy against HPV16/18 and preventing 63.6% of other high risk HPV infections

*Screening scenarios provide screening ages, except 3/5 (6/10)-yearly represents screening every 3(6) years aged 25-49 years and every 5(10) years aged 50-64 years.
